# The recombination-cold region as an epidemiological marker of recombinogenic opportunistic pathogen *Mycobacterium avium*

**DOI:** 10.1186/s12864-019-6078-2

**Published:** 2019-10-17

**Authors:** Hirokazu Yano, Haruo Suzuki, Fumito Maruyama, Tomotada Iwamoto

**Affiliations:** 1 Graduate School of Life Sciences, Tohoku University, Katahira, Aoba-ku, Sendai, Japan; 2 Faculty of Environment and Information Studies, Keio University, Fujisawa, Japan; 3 Office of Industry-Academia-Government and Community Collaboration, Hiroshima University, Hiroshima, Japan; 4 Department of Infectious Diseases, Kobe Institute of Health, Kobe, Japan

**Keywords:** MAC, NTM, VNTR, MLVA, Genotyping, SNP, Genetic population structure, BAPS, Mycobacterium, Marker gene

## Abstract

**Background:**

The rapid identification of lineage remains a challenge in the genotyping of clinical isolates of recombinogenic pathogens. The chromosome of *Mycobacterium avium *subsp. *hominissuis* (MAH), an agent of *Mycobacterium avium* complex (MAC) lung disease, is often mosaic and is composed of chromosomal segments originating from different lineages. This makes it difficult to infer the MAH lineage in a simple experimental set-up. To overcome this difficulty, we sought to identify chromosomal marker genes containing lineage-specific alleles by genome data mining.

**Results:**

We conducted genetic population structure analysis, phylogenetic analysis, and a survey of historical recombination using data from 125 global MAH isolates. Six MAH lineages (EA1, EA2, SC1, SC2, SC3, and SC4) were identified in the current dataset. One P-450 gene (locus_tag MAH_0788/MAV_0940) in the recombination-cold region was found to have multiple alleles that could discriminate five lineages. By combining the information about allele type from one additional gene, the six MAH lineages as well as other *M. avium* subspecies were distinguishable. A recombination-cold region of 116 kb contains an insertion hotspot and is flanked by a mammalian cell-entry protein operon where allelic variants have previously been reported to occur. Hence, we speculate that the acquisition of lineage- or strain-specific insertions has introduced homology breaks in the chromosome, thereby reducing the chance of interlineage recombination.

**Conclusions:**

The allele types of the newly identified marker genes can be used to predict major lineages of *M. avium.* The single nucleotide polymorphism typing approach targeting multiallelic loci in recombination-cold regions will facilitate the epidemiological study of MAC, and may also be useful for equivalent studies of other nontuberculous mycobacteria potentially carrying mosaic genomes.

## Background

*Mycobacterium avium* complex (MAC), consisting of *M. avium*, *M. intracellualare*, and several rarely reported taxa, is the most common non-tuberculous mycobacterial group causing pulmonary disease in Asia and Europe [1, 2]. Among MACs in a human clinical setting, *M. avium* subsp. *hominissuis* (MAH) is currently the most frequently encountered subspecies [1, 2]. MAC is thought to reside in natural environments and human-built architecture including potable water systems and bathroom biofilm [3–10]. MAC is generally difficult to eradicate by antibiotic treatment, and its infection mechanisms, lineage-specific phenotypic characteristics, and pattern of short- and long-term genetic changes are very poorly understood.

In mycobacterial infections, the rapid identification of the pathogenic lineage is important because the lineage can be associated with virulence level and antibiotic susceptibility [11–14]. To study the transmission pattern of pathogens, a variety of fragment length-based genotyping approaches have been developed. One representative is multiple-locus variable number tandem repeat analysis (MLVA), which uses information about the repeat number at several (4–19, depending on the purpose) highly variable repeat loci to determine the genetic relatedness of the isolates [15–17]. Insertion sequence fingerprinting is another representative approach [16, 18, 19]. Because both techniques assess the fragment length of highly variable loci with reversible repeat numbers, the dissimilarity of fragment length patterns may not reflect the relatedness of the remaining genome, which gradually diversifies, accumulating mutations and recombinations, during the long-term evolution of the species. Therefore, the single nucleotide polymorphism (SNP)-based approach [20] is better suited for lineage inference.

A whole genome epidemiological study of MAH has recently started [21–23]. We previously revealed the presence of multiple MAH lineages within a single country, most notably Japan, and the occurrence of extensive interlineage recombination on the chromosome [21]. Thus, clonal expansion is not the major mechanism underlying the local diversification of MAH. The population structure of global MAH isolates inferred from the 14-loci MLVA data was roughly consistent with the distribution of MAH lineages inferred from core genome SNP data [21]. While this suggests that MLVA is still useful for MAH lineage identification, MLVA for more than 10 loci can be labor-intensive, occasionally giving rise to vague interpretation results, so may not be cost-effective to infer the lineage of MAH isolates.

The minimum SNP approach, which only assesses SNPs on particular genes, has proved effective for rapid subspecies identification of MAC [24, 25] and *Mycobacterium abscessus* [26]. While this approach can reduce labor costs and is free from result ambiguity, it remains unclear whether the nucleotide diversity in the currently used marker genes contains sufficient information to distinguish between lineages despite the occurrence of chromosomal recombination. Because particular genetic population groups of MAH are associated with livestock disease in Japan [27, 28], it is important to find a genetic marker for the rapid identification of MAH lineages.

To this end, we newly defined six major MAH lineages using increased genome data available since February 2018 based on a population genetics method. By focusing on recombination-free or ‘cold’ regions, we screened chromosomal loci containing lineage-specific alleles. This recombination-cold gene typing approach could facilitate future epidemiological studies of MAC.

## Results

### Detection of *M. avium* lineages

**Fig. 1:**
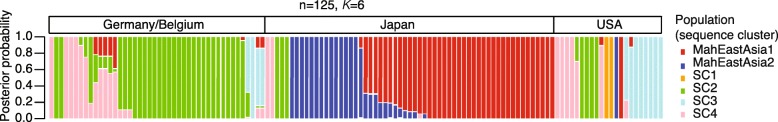
Admixture analysis for the genomes of 125 global isolates. Genetic populations and admixture were inferred using BAPS version 6.0 [31]

MAH lineages in the global population were inferred using 48,972 filtered polymorphic sites detected in 125 genomes, which have been available in the PATRIC database [29] since February 2018 (Additional file 1). To obtain core genome SNPs, we used the genome of the Japanese isolate TH135 as a reference. Genetic population structure was first inferred using BAPS software [30]. With the present genome SNP data, separation into six subpopulations (sequence cluster) was suggested to be optimal according to the maximum log likelihood score in the mixture analysis of BAPS (*k* = 6, log(ml) = − 1,588,774.4374; *k* = 7, log(ml) = − 1,596,789.585; *k* = 8, log(ml) = − 1,609,521.5204). In the following admixture analysis (Fig. 1), 28 isolates were suggested to possess admixed genomes consisting of segments originating from distinct genetic populations: SC1, SC2, SC3, SC4, MahEastAsia1 (hereafter referred to as EA1), and MahEastAsia2 (EA2). An SC4-equivalant population was previously identified as part of the SC2 population [21]. Consistent with this earlier study performed with fewer data, EA1 and EA2 signals predominated in the genomes of Japanese (clinical) isolates, but not in isolates from Germany, Belgium, or the USA.

**Fig. 2:**
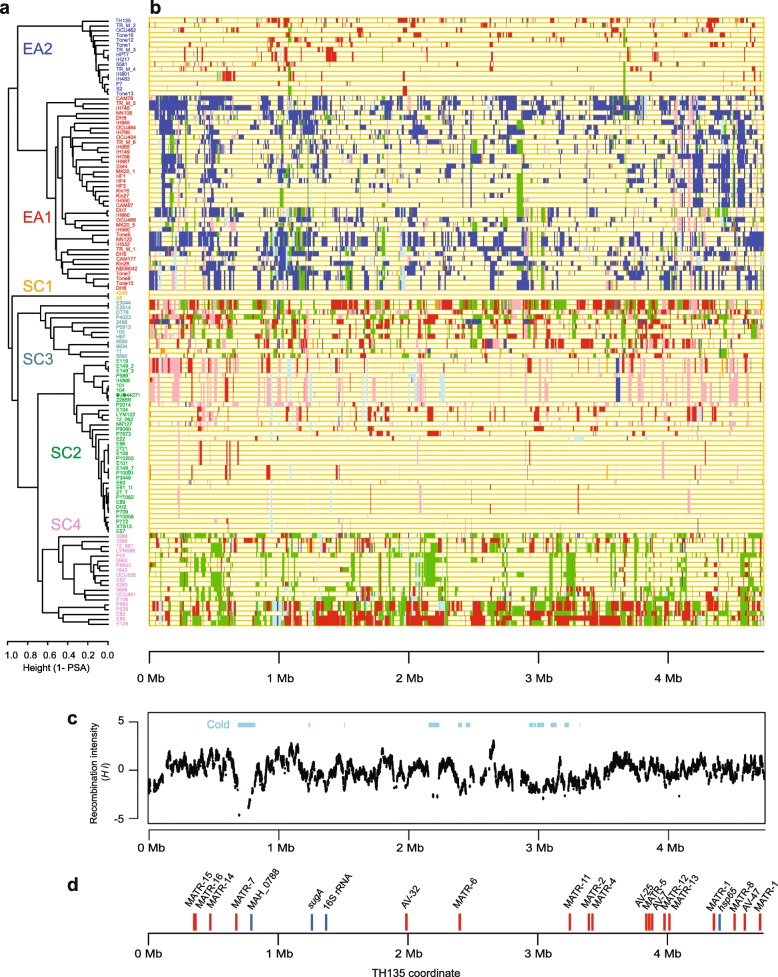
MAH lineages in global MAH population. **a** Complete linkage clustering of 125 isolates based on genetic linkage. The distance is based on 1 – PSA (see main text). Isolate names are shown as a distinct color per lineage, **b** Visualization of recent imports inferred by fastGEAR [32]. Color of the chromosomal chunks indicates donor lineage of the chunk, **c** Recombination intensity inferred by OrderedPainting [33]. Recombination-cold regions are shown s horizontal bars, **d** Genomic location of known and new marker genes. Red: VNTR locus, Blue: genes used in SNP-based typing. Marker gene information was collected from [16, 24, 34]

The MAH lineage was more accurately inferred using fastGEAR, taking into account genetic linkage [32]. This analysis detected six lineages (Fig. 2), which were equivalent to the sequence clusters predicted by BAPS (Fig. 1). Thus, we used BAPS sequence cluster names to refer to the MAH lineages. Recent recombinations were detected in all genomes except for the two members of SC1. The number of detected recent recombination tracts and total import lengths were generally large in EA1 (median tracts = 122, median total import length = 1.35 Mbp), SC3 (median tracts = 129, median total length = 1.5 Mbp), and SC4 (median tracts = 118, median total length = 1.4 Mbp), while they were small in EA2 (median tracts = 36, median total length = 0.31 Mbp) and SC2 (median tracts = 23, median total length = 0.16 Mbp) (Additional file 2).

### Genes holding lineage-specific alleles

To search for genes carrying lineage-specific alleles, we next focused on a subset of genes located near recombination-cold regions. Recombination hot/cold regions were estimated using OrderedPainting software which evaluates the local recombination intensity relative to the genome average (Fig. 2C) [33]. Codon alignments of 138 core genes located within recombination-cold regions (*Hi* score < − 2.0) were analyzed for (i) nucleotide diversity, (ii) haplotype diversity (probability of allele difference) [35], and (iii) the number of alleles in the alignment. The filtering based on their values (see Method) identified five marker gene candidates, including orthologs of strain TH135 gene MAH_0771 (TH135 coordinate 772,960), MAH_0766 (TH135 coordinate 767,567), MAH_0788 (TH135 coordinate 795,348), MAH_0809 (TH135 coordinate 813,897), and MAH_2714 (TH135 coordinate: 2,965,182).

**Fig. 3:**
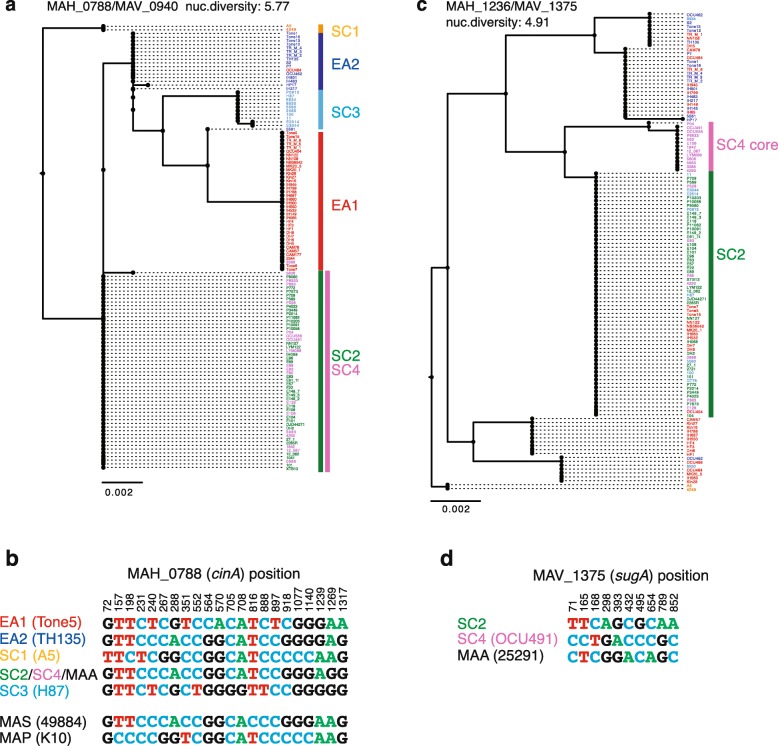
Chromosomal loci carrying lineage-specific alleles. **a** Maximum likelihood tree based on the MAH_0788 ortholog alignment. GTR + G was used as the model. Truncated entries were removed upon construction of the tree, **b** Polymorphic sites in the alignment of MAH_0788 orthologs. Variation was not detected among the members of SC2 and SC4, **c** Maximum likelihood tree based on the alignment of the MAV_1375 ortholog. GTR + G was used as the model, **d** Selected polymorphic sites in the alignment of the MAV_1375 ortholog

Members of each of the five major lineages (SC1, SC2 plus SC4, SC3, EA1, and EA2) tended to cluster at one node in the trees of MAH_0766 (Mah104 locus_tag MAV_0925; product name of ‘NADH dehydrogenase’), MAH_0771 (Mah104 locus_tag MAV_0930; product name of ‘amidohydrolase’), MAH_0788 (Mah104 locus_tag MAV_0940; *cinA* gene, product name of ‘1,8-cineole 2-endo-monooxygenase’ or ‘cytochrome P-450’), and MAH_0809 (Mah104 locus_tag MAV_0960, product name of ‘hypothetical protein’), but not in MAH_2714 (Additional file 3). MAH_0766, MAH_0771, MAH_0788 are located in the recombination-cold region at chromosome coordinates 683,022 to 799,327 (*Hi*: − 4.6 to − 2.0) (Fig. 2d). SC2 and SC4 share an allele in these three genes (Fig. 3a). In the MAH_0788 gene tree, an exception was seen in only three isolates, OCU464, 3388, and 10–5581 (denoted as 5581 in the phylogenetic tree). The *hsp65* ortholog (a *groEL* paralog) has been used as a subspecies identification marker in the genotyping of MAC isolates [24]. In the *hsp65* gene tree, the isolates were not clustered per lineage (Additional file 3). This may reflect a lack of sequence diversity or the occurrence of inter-lineage recombination. The MAH_0788 alignment contained 21 polymorphic sites, characterizing five representative alleles (Fig. 3b). Those sites could be used to assign isolates into five major lineages.

We next investigated whether the allele types of the MAH_0788 ortholog could distinguish each MAH lineage from other MAC subspecies: namely, MAP, MAA, and MAS. Sequence alignment revealed that the alleles of MAP and MAS are respectively unique, while the allele is shared between MAA and SC2/SC4 (Additional file 4, Fig. 3b).

Because the members of SC2 and SC4 showed identical sequences in the MAH_0788 ortholog sequence, the MAH_0788 ortholog nucleotide sequence alone cannot distinguish between SC2 and SC4. Furthermore, SC4 members do not carry unique SNPs in *hsp65*. Therefore, we screened recombination-cold regions based on the genome alignment of the SC2/SC4 data set, which contained 31,598 polymorphic sites. For this data set, we used Gubbins software [36], which produces recombination-free SNPs alignments and simultaneously infers phylogenetic trees. Except for five highly admixed members of SC4 (strains P528, P883, E83, E88, and E128), 14 core members of SC4 were clustered in one branch in the maximum likelihood tree (Additional file 5), suggesting that SNPs unique to these members were present. After manual screening for recombination-free regions, two loci, MAV_1375 (TH135 locus_tag MAH_1236, *sugA* gene, product name of ‘trehalose ABC transporter’) (Fig. 3C) and MAV_2820 (TH135 locus_tag MAH_2466, product name of ‘short chain dehydrogenase’) (Additional file 3), were found to contain SNP tracts, each giving rise to two types of alleles that were unique to SC4 core members. Furthermore, in the MAH_1236/MAV_1375 locus, the MAA allele was distinguishable from SC2 and SC4 alleles (Fig. 3d). These results together suggested that allele types in two chromosomal loci, the *cinA* gene encoding P-450 (locus_tags: MAH_0788, MAV_0940) and the *sugA* gene encoding the trehalose ABC transporter (locus tags: MAH_1236, MAV_1375), could additively inform the core genome lineage (or association of BAPS subpopulations) of MAH isolates as well as *M. avium* subspecies.

### Features around the recombination-cold region

Rare occurrences of historical recombination could be caused by mechanistic bias for DNA transfer or homologous recombination. Therefore, to obtain insights into the origin of the recombination-cold region, we compared the structures of these regions in four completely sequenced strains (TH135, OCU464, Mah104, and H87), each from a different lineage. The 116 kb recombination-cold region (*Hi* score < − 2.0) includes a previously reported insertion hotspot containing clustered tRNA genes and an East Asian lineage-unique segment (Additional file 6, panels A and B). This region is flanked by a mammalian cell entry protein (MCE) operon where allelic variants have previously been reported [21, 37]. Therefore, this recombination-cold region may lack targeting by homologous recombination through homology breaks introduced by lineage- or strain-specific insertions occurring at relatively close distances. We noted that chromosomal segments containing MAH_0788, MAH0771, and MAH_0766 orthologs are present in other NTM species (i.e., *M. chimaera*, *M. intracellulare*, *M. kansasii*, and *M. marinum*)*,* but the equivalent segments are missing in *M. ulcerans*, *M. abscessus*, *M. tuberculosis*, *M. canettii*, and *M. smegmatis* (Additional file 6, panels C and D).

## Discussion

The genotyping of clinical isolates is the initial step for treatment planning and the estimation of infection sources. Because MAC-associated diseases are increasing worldwide [1, 2], it is important to establish common and user-friendly methods for *M. avium* genotyping. In the current genome data collection, we identified six lineages, taking genetic linkage into account. Two of these, EA1 (with a large number of recombinations) and EA2 (with a small number of recombinations), were predominantly found in clinical isolates in Japan, and two others, SC2 (with a small number of recombinations) and SC4 (with a large number of recombinations), were commonly found in clinical and environmental isolates in Germany. SC3 and SC1 have also frequently been found in the USA (Table 1). Therefore, the genome lineage may reflect the geographic location of the isolate ancestor. An atypical situation was seen in pig isolates in Japan (OCU556 and OCU491), which belong to the newly defined SC4 lineage. We speculate that this could reflect the immigration of SC4 members as hundreds of pigs are imported from Europe, the USA, and Canada every year for breeding purpose according to the annual report from ministry of agriculture, forestry and fisheries of Japan [41].

**Table 1:** Features of MAH lineages

Lineage	Major isolation countries^1^	Host or niche^2^	Notable feature of the chromosome
EA1	Japan, Korea	Human adult^3^, bathroom^5^	Highly mosaic
EA2	Japan, Korea	Human adult^3^, bathroom^5^	Relatively few imports, inversion^4^
SC1	USA	Little information	Little information
SC2	Germany, Belgium, the Netherlands, Russia, USA, Japan (pig isolates)	Human adult^3^ and child, soil, dust, pig	Relatively few imports
SC3	USA, Germany	Animals, water, soil, human	Highly mosaic
SC4	Germany, Belgium, the Netherlands, Russia, USA, Japan (pig isolates)	Animals, soil, dust, human adult and child	Close relative of SC2, highly mosaic

1. Apart from the present study, the lineages of human isolates in Korea, Russia, and the Netherlands, and isolates from bathrooms and pigs in Japan were deduced previously using BAPS mixture analysis based on 14 loci MLVA data [21, 27, 38, 39]

2. Hosts and niches of sequenced isolates are shown in Additional file 1: Table S1

3. Uchiya et al [40] reported a significant difference in antibiotic susceptibility between EA1, EA2, and SC2 human isolates

4. Uchiya et al [37] reported a difference in chromosome structure between TH135 (EA2) and Mah104 (SC2)

5. Arikawa et al [10] reported the presence of MAH in the bathrooms of healthy volunteers

The current MAC phenotype–lineage relationship is rather vague, perhaps because lineage-informative loci have been undetected until now and it has been difficult to set up experimental research to address this problem. Recently, Uchiya et al. [40] demonstrated that clinical isolates belonging to East Asian lineages are more resistant to antibiotics than those of the SC2 lineage. The SC4 lineage is associated with animals, particularly lymphadenitis in the digestive tracts of pigs [27], while SC2 is common in adult pulmonary infection and child lymphadenitis in Europe [22]. The MAC lung disease incidence rate is highest in Japan among industrialized countries [2], suggesting that EA1 and EA2 lineages may be more virulent than others. Rapid identification of MAC taxa at the lineage level is likely to be required in the near future, particularly as multiple MAH lineages are now known to be present in every country (Fig. 1 and Additional file 1: Table S1).

In MAC infection diagnosis, the most commonly used flow is the isolation of *Mycobacterium* colonies on the selection plate and 16S rRNA gene typing, followed by *hsp65* (*groEL* paralog) typing. This enables the identification of MAC taxa at the subspecies level, but not at the lineage level (Additional file 3). The 14 loci MLVA remains an effective approach to infer lineage [21], perhaps because previously proposed MLVA loci are scattered throughout the genome (Fig. 2D), but this technique is not labor effective or a good method principle. Therefore, this study aimed to establish a minimum SNP approach to identify the MAH lineage. One locus, MAH_0788 (a P-450 gene), was shown to be located in the recombination-cold region and to contain allelic variations capable of distinguishing between five major MAH lineages. The P-450 gene even discriminated MAH from MAP and MAS. Although the allele of this P-450 gene is shared among SC2, SC4, and MAA, additional information in *sugA* (MAH_1236/MAV_1375), located in the recombination-cold region between the SC2 and SC4 populations, enabled the distinction between SC2, SC4, and MAA. As lineage indicative SNPs are distributed in close proximity within MAH_0788, PCR amplification and Sanger sequencing of an approximately 600 bp-long segment covering positions 705 to 1269 will discriminate the five major lineages. Similarly, sequencing a short segment in MAV_1375, from position 78 to 168, will distinguish SC2 from SC4. We have also listed the secondary marker genes, the MAH_0771 locus for five-lineage distinction, and the MAV_2820 (MAH_2466) locus for SC2 and SC4 distinction (Additional file 3). SNPs in these secondary loci can be jointly used to predict the lineage. Line probe assay tools have already been developed for *Mycobacterium* species and subspecies identification [42]. Therefore, the lineage marker gene can also be used as a probe in the line probe assay for the simultaneous identification of subspecies and lineage.

The recombination cold-region identified in the present study contains previously reported clustered tRNA genes that potentially serve as an insertion hotspot [8]. This region contains an insert variation among European isolates belonging to the SC2 and SC4 lineages [8, 22]. A similar hotspot has been found in *Klebsiella pneumoniae* [43]. We newly identified a DNA sequence insertion into tRNA-Phe in strain OCU464 (EA1), and insertions into both tRNA-Phe and tRNA-Lys in strain H87 (SC3) (Additional file 6). As these inserts each encode an integrase (tyrosine recombinase) they are likely to be mobile elements (e.g. genomic islands or prophage) that target tRNA [44]. It is reported that mobile element integration increases recombination within neighboring core genes in *Staphylococcus aureus* [45]*.* Moreover, a systematic survey of 80 bacterial species revealed that core genes flanking insertion hotspots are more targeted by homologous recombination than other parts of the core genome [46]. However, this is not the case for the above described hotspot in *M. avium.* We speculate that the maintenance of the recombination-cold region in *M. avium* reflects the reduced chance of homologous recombination occurring because of the acquisition of a lineage-specific insertion and lineage-specific allele cluster in close proximity during the historic local diversification of MAH. Another non-mutually exclusive hypothesis for the infrequent recombination we observed relates to the selection of co-evolving genes in the operonic structure. Such genes might be *cinA* (cytochrome P-450) and its neighboring genes, *subB* (ferredoxin) and *phbB* (acetoacetyl-CoA reductase), as the region containing these genes is conserved in at least five NTM species for which the complete chromosome sequences are available. It remains to be determined whether a recombination-free or cold region also exists in the genomes of other mycobacterial species, which are reported to possess mosaic genomes [47–49].

## Conclusions

Six MAH lineages were identified in the currently available data of global MAH isolates. We found that the P-450 gene in the recombination-cold region contained allelic variations that can distinguish between five major lineages, despite the genome-wide occurrence of interlineage recombination. Furthermore, allele information of one additional gene allowed the distinction between six lineages. We propose the minimum-SNP typing approach focusing on a multiallelic locus in a recombination-cold region as a novel epidemiological genotyping method that can easily be applied to all bacteria without obvious virulence markers.

## Methods

### Dataset

Genomic and associated metadata of 125 MAH isolates were retrieved from the PATRIC database in February 2018 [29]. Information about the isolates is shown in Additional file 1. This dataset includes nine complete and 116 draft genomes. As a reference sequence for *M. avium* subsp. *avium* (MAA), subsp. *silvaticum* (MAS), and subsp. *paratuberculosis* (MAP), genome sequences of a type or well-known strain ATCC 25291, ATCC 49884, and K-10 were obtained from RefSeq (accession nos. NZ_ACFI00000000.1, NZ_AYOC01000000.1, and NC_002944.2, respectively). We used the sequences from strains AH16 (GenBank accession no. CP012885.1), ATCC12478 (CP006835.1), ATCC 13950 (RefSeq accession no. NC_016946.1), M (NC_010612.1), Agy99 (NC_008611.1), ATCC 19977 (NC_010397.1), H37Rv (NC_000962.2), CIPT 140010059 (NC_015848.1), and MC2 155 (NC_008596.1) as the reference sequences for *M. chimaera*, *M. kansasii*, *M. intracellulare*, *M. marinum*, *M. ulcerans*, *M. abscessus*, *M. tuberculosis*, *M. canettii*, and *M. smegmatis.*

### Lineage assignment and recombination detection

The *M. avium* lineage was defined using a non-phylogenetic method, namely BAPS and its related program, fastGEAR, which clusters individuals based on genetic linkage patterns after detecting recombinations between BAPS subpopulation groups called sequence clusters (SCs) [30, 32]. BAPS software predicts the optimal partition of a population into random mating units without using a phylogenetic model) [30]. It was chosen to infer clusters of closely related MAH isolates because many isolates have mosaic genomes so a population genetics method used for sexual organisms is suitable for lineage inference, and because the same analysis can be performed with MLVA data which have widely been used for epidemiological studies of MAC [27, 50], allowing the association of results with those based on an MLVA dataset [21]. To generate input files for this analysis, polymorphic sites were detected by aligning draft or complete genome sequences with the complete genome sequence of strain TH135 (Japanese isolate) using Parsnp v 1.2 software [51]. Polymorphic sites with flags (SNPs in < 200 bp locally collinear block, or SNPs in 100 bp windows containing > 20 Indels, and sites containing N) were not used for subsequent analysis. A total of 48,972 filtered polymorphic sites were detected in the core genome of the 125-genome data set. Haplotype information of the 48,972 filtered polymorphic sites was used for sequence cluster (SC) identification by BAPS (v 6) mixture analysis. The BAPS input file was generated by converting the vcf file of the Parsnp output into BAPS format using PGDSpider format-converter [52]. The output file of BAPS mixture analysis (clustering of individuals) was directly used for the following admixture analysis in BAPS [31] to infer the subpopulations that donated SNPs to individuals (Fig. 1).

Filtered polymorphic sites were combined with intervening reference genome sequences in a multi-fasta format, and used as input for fastGEAR (running on MATLAB compiler runtime v 9.0.1). In the lineage estimation by fastGEAR, the distance between two isolates is defined as 1 – the proportion of chromosomal fragments sharing the ancestry (PSA), and the lineage is determined as a result of hierarchical clustering [32]. fastGEAR predicts two types of recombination separately: (i) recent recombination, which is interlineage recombination where donor–recipient relationships can be inferred parsimoniously; and (ii) ancestral recombination between the common ancestors of each lineage, where donor–recipient relationships cannot be inferred. Upon construction of a PSA tree, both recombinations are taken into account, i.e. part of the ancestral recombination tracts can be overwritten with recent recombination tracts when assigning the ancestry of the chromosomal segment.

To evaluate recombination frequency throughout the chromosome, we used the *Hi* statistic known as the realized recombination rate (intensity of recombination relative to genome average) inferred by OrderedPainting [53]. In the present study, the stretches of 30 polymorphic sites with median *Hi* < − 2.0 (sites ranked in the bottom 2.55%) were regarded as recombination-cold regions, and were used to screen for single copy core orthologous genes (see below) embedded within these regions.

A total of 31,598 filtered polymorphic sites were obtained for the core genome of 54 isolates consisting of only SC2 and SC4 members, using the complete genome sequence of strain Mah104 as a reference. Filtered polymorphic sites of this SC2/SC4 dataset, obtained as described above, were used for phylogenetic analysis and recombination detection using Gubbins software [36]. This software is more suitable for the genealogical analysis of a population consisting of closely-related haplotypes than fastGEAR, and is useful as it generates multiple sequence alignment of recombination-free polymorphic sites [36]. Recombination-cold regions that can distinguish between SC2 and SC4 were screened manually using fasta and vcf files of the Gubbins outputs.

### Core gene screening

To identify core genes in the MAH population, homologous gene clustering was performed using the CD-HIT algorithm implemented in Roary v 3.7.0 software [54]. The 125 MAH genomes were re-annotated in-house using PROKKA v 1.2 software [55], and GFF3 files were used as an input for Roary. Nucleotide sequences of core genes were obtained using Roary with -cd 95 -e --mafft -n -z –f options, followed by a collection of gene alignments containing only one entry from each genome. The -cd 95 option handles genes at the 95% conservation level as a core. This option was used because most genomes in the 125-genome dataset were drafts so more strict criteria, such as 100% conservation levels, may have missed true core genes. The minimum percentage identity for blastp was 95%. Potentially truncated entries, shorter than the median length for all entries, were filtered out from each alignment. Filtered codon alignments with fewer than 100 entries were not considered marker gene candidates. Codon alignments were then generated using PRANK v.150803 software with the ‘–codon’ option, using mafft -generated alignments as starting files [56, 57].

### Genetic diversity and phylogenetic analyses

Codon alignments were analyzed to calculate the average pairwise nucleotide diversity, haplotype diversity, and number of alleles in the alignment using the PopGenome package of R [58]. Genes for lineage markers should have a sufficiently high genetic diversity to detect differences between lineages, but the diversity should not be too high within each lineage. Marker gene candidates that could distinguish among five lineages based on allele sequences were first screened to meet the following criteria: average pairwise nucleotide diversity > 5.0; probability of haplotype (allele) differences (haplotype diversity *H* [35]) > 0.6 and < 0.8; and number of alleles > 5 and < 12. If we assume the presence of six lineages in population and the following distribution of individuals: SC1, 2; SC3, 12; SC2, 36; SC4, 19; EA2, 16; EA1, 40, then the ideal number of allele in population would be six, whereas haplotype diversity would be 0.7719. If we assume SC2 and SC4 as one lineage (total number of lineage is five), the ideal number of allele in population would be five, whereas haplotype diversity would be 0.6837.

The codon alignments of marker gene candidates were further converted to Phylip format using a Perl script, and used to infer the maximum likelihood phylogenetic tree in PhyML based on the GTR + G model [59]. The allele of strain A5 was set as the outgroup when constructing phylogenetic trees. Trees were visualized using Figtree v 1.4 software (http://tree.bio.ed.ac.uk/software/figtree/). The gene trees where most of the isolates of one lineage clustered on a single node were selected as the trees of the lineage marker gene.

### Genome structure comparison

The structures of completely sequenced chromosomes were compared and visualized in GenomeMatcher software [60] using the blastn or blastp algorithm.

## Supplementary information

**Additional file 1.** Information of data sources used in this study.

**Additional file 2.** Difference in the number and total fragment lengths of recent recombination events between MAH lineages.

**Additional file 3.** Phylogenetic trees based on codon alignments of marker gene candidates and *hsp65*. (i) MAH_0788/MAV_0940 ortholog, (ii) MAH_0771/MAV_0930 ortholog, (iii) MAH_0766/MAV_0925 ortholog, (iv) MAA_0809/MAV_0960 ortholog, (v) MAH_2714/ MAV_2410 ortholog (inadequate marker gene), (vi) *hsp65* ortholog, (vii) MAV_1375 ortholog, (viii) MAV_2820 ortholog Trees were constructed using the GTR + G model in PhylML with 100 times bootstrapping run. Values by the branch indicate the number of bootstrap supports. Allele of strain A5 was used as an out group for the analysis in panels i–vi. The out group was not used for tree construction in panels vii and viii. Alignment information is shown by the tree as follows: n.allele, number of alleles in the alignment (population); nuc.diversity, average pairwise nucleotide diversity (number of site differences) in the alignment; hap.diversity, probability of haplotype (allele) differences in the alignment; alignment length, length of alignment without gaps.

**Additional file 4.** Alignment of lineage-specific alleles. (A) MAH_0788/MAV_0940 locus (*cinA*/P-450 gene). (B) MAH_1236/MAV_1375 locus (*sugA* gene). Polymorphic sites were indicated by distinct color. Sites used to distinguish among *M. avium* lineages were indicated by asterisks under the alignment.

**Additional file 5.** Phylogeny inference of SC2 and SC4 members by Gubbins. (Left) Phylogenetic tree based on recombination-tract free alignments. Scale bar indicate the number of SNPs. SC2 members were shown in green, while SC4 members were shown in magenta. (Right) Location of recombination tracts. Recombination tracts introduced in internal braches were shown in red. Recombination tracts unique to terminal branch is shown in blue. (PDF 822 kb)

**Additional file 6.** Insertions near the recombination-cold region. (A) Similarity between two chromosomes. Locations of the recombination-cold region and the MCE operon locus 3 are indicated by white horizontal lines. The similarity between two genomes was determined by the blastn algorithm implemented in GenomeMatcher software [60]. Genomic positions are shown by strain names. (B) Insertions in the tRNA gene cluster. Green pentagons indicate the tRNA gene. Yellow pentagons or circles indicate integrase (a tyrosine recombinase, *int*) or excisionase (*xis*). The insert in strain H87 contain 31 copies of recombinase genes. (C) Conservation of marker genes in the MAH recombination-cold region in *M. chimaera*, *M. intracellulare*, *M. marinum*, and *M. kansasii*. (D) Absence of marker genes in *M. abscessus, M. ulcerans, M. tuberculosis*, *M. canettii*, and *M. smegmatis*. (PDF 7032 kb)

## Data Availability

Nucleotide sequence data analyzed during this study are available from public database accession numbers listed in Additional file 1. Software input and output files generated in study is available from corresponding author(s).

## Abbreviations

MAC: *Mycobacterium avium* complex
MAH: *Mycobacterium avium* subsp. *hominissuis*
MLVA: Multiple-locus variable number tandem repeat analysis
SC: Sequence cluster
SNP: Single nucleotide polymorphism
VNTR: Variable-number tandem-repeat
