# Supplementary material for: The recombination-cold region as an epidemiological marker of recombinogenic opportunistic pathogen Mycobacterium avium
Source: BMC Genomics. 2019 Oct 17;20:752. doi: 10.1186/s12864-019-6078-2 (PMC6798384; doi:10.1186/s12864-019-6078-2)
Supplement: Supplementary file 2 — Additional file 2. Difference in the number and total fragment lengths of recent recombination events between MAH lineages. [file 12864_2019_6078_MOESM2_ESM.pdf]

Additional file 2

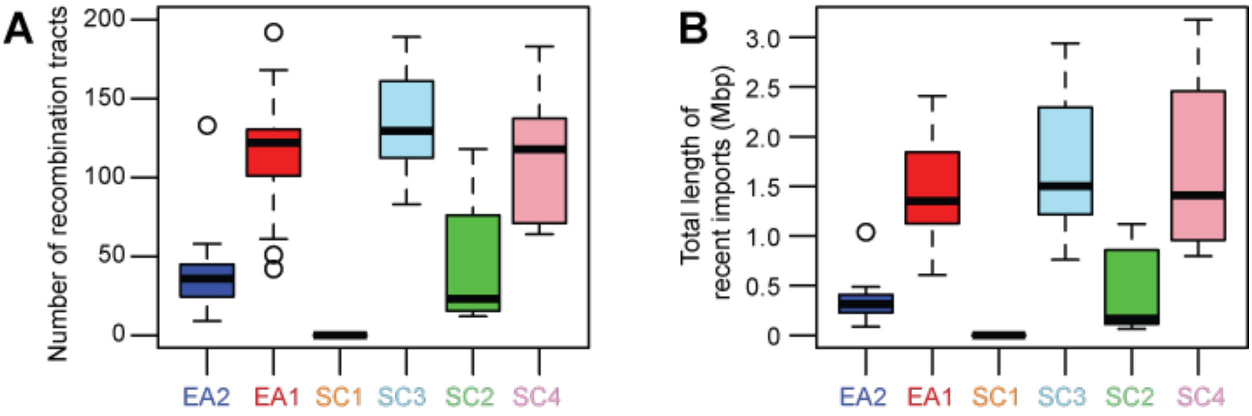


**Difference in the number and total fragment lengths of recent recombination events between MAH lineages.** The number of the lineage member is as follows: SC1, 2; SC3, 12; SC2, 36; SC4, 19; EA2, 16; EA1, 40. (A) number, (B) total fragment lengths.
