# Supplementary material for: The recombination-cold region as an epidemiological marker of recombinogenic opportunistic pathogen Mycobacterium avium
Source: BMC Genomics. 2019 Oct 17;20:752. doi: 10.1186/s12864-019-6078-2 (PMC6798384; doi:10.1186/s12864-019-6078-2)

The recombination-cold region as an epidemiological marker of recombinogenic opportunistic pathogen *Mycobacterium avium*

Hirokazu Yano[1], Haruo Suzuki[2], Fumito Maruyama[3], Tomotada Iwamoto[4]

1. Graduate School of Life Sciences, Tohoku University. Katahira, Aoba-ku, Sendai, Japan
2. Faculty of environment and information studies, Keio University, Fujisawa, Japan
3. Office of Industry-Academia-Government and Community Collaboration, Hiroshima University, Japan.
4. Kobe Institute of Health, Kobe, Japan

Contents of additional file 3

Phylogenetic trees based on codon alignments of marker gene candidates and the *hsp65* gene.

(i) MAH_0788/MAV_0940 ortholog,
(ii) MAH_0771/MAV_0930 ortholog,
(iii) MAH_0766/MAV_0925 ortholog
(iv) MAH_0809/MAV_0960 ortholog,
(v) MAH_2714/ MAV_2410 ortholog (inadequate marker gene),
(vi) *hsp65* ortholog,
(vii) MAV_1375 ortholog,
(viii) MAV_2820 ortholog

Trees were constructed using GTR+G model in PhyIML with 100 times bootstrapping run. Values by the branch indicate the number of bootstrap support. Allele of strain A5 is used as out group for the analysis in panels i-vi. Out group was not used for tree construction of panels vii and viii. Alignment information is also shown by the tree as follows: n.allele, number of alleles in the alignment (population); nuc.diversity, average pairwise nucleotide diversity (number of site differences) in the alignment; hap.diversity, probability of haplotype (allele) differences in the alignment; alignment length, length of alignment without gaps.

Additional file 3

(i) MAH_0788

locus_tag: MAH_0788 in strain TH135, MAV_0940 in strain 104
gene name: cinA-1
product name: 1,8-cineole 2-endo-monooxygenase (P-450)
nuc. diversity: 5.7719
hap. diversity: 0.6947
n. allele: 9
TH135 coodinate: 795,348
alignment length: 1,362 bp

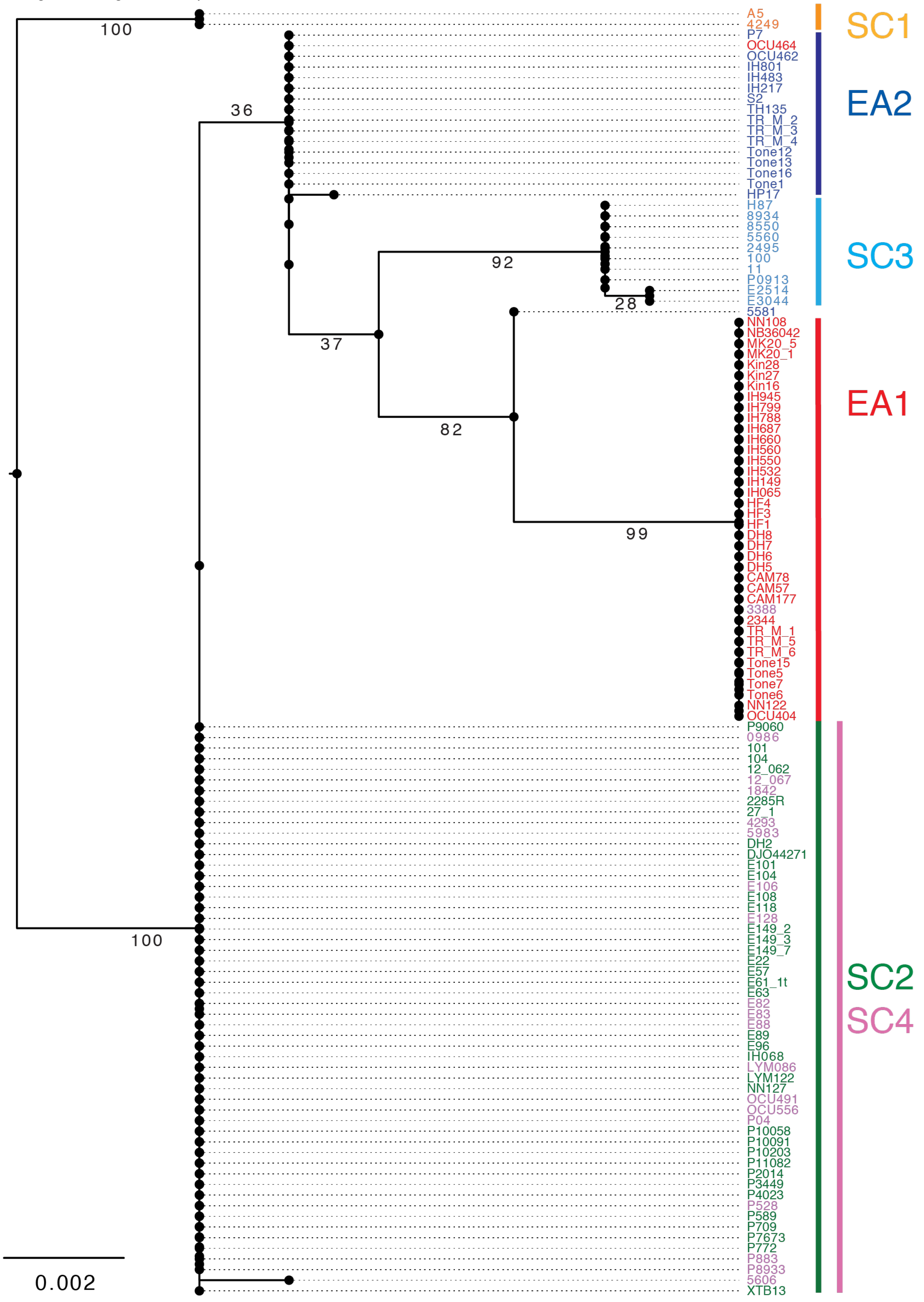

Additional file 3

(ii) MAH_0771

locus_tag: MAH_0771 in strain TH135;
MAV_0930 in strain 104
gene name: not assigned
product name: amidohydrolase
nuc. diversity: 9.9832
hap. diversity: 0.6999
n. allele: 7
TH135 coodinate: 772,960
alignment length: 1,098 bp

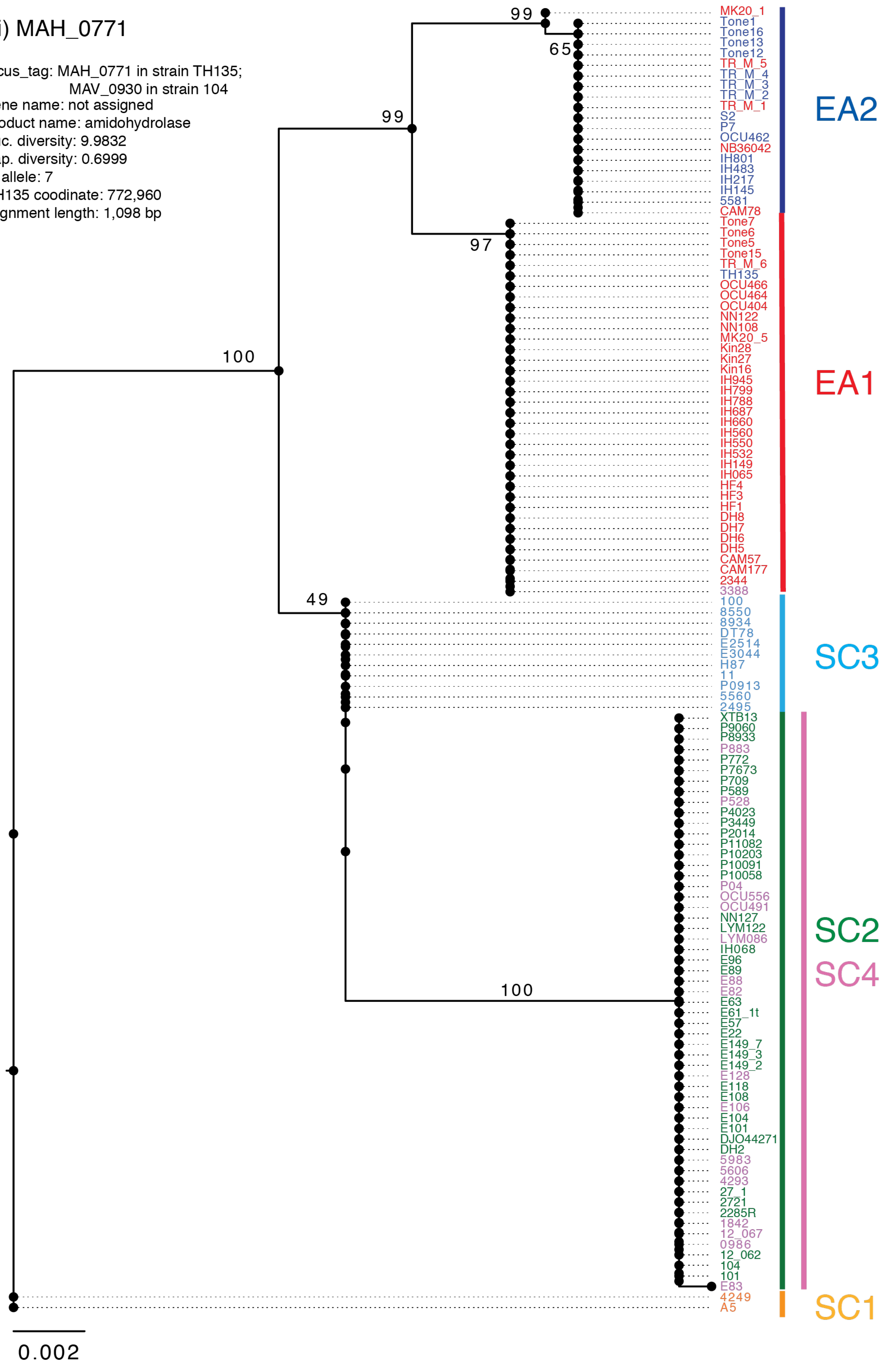

Additional file 3

(iii) MAH_0766

locus tag: MAH_0766 in strain TH135; MAV_0925 in strain 104
gene name: *nuoF*
product name: NADH dehydrogenase
nuc.diversity: 7.9939
hap.diversity: 0.7110
n. allele: 8
TH135 coordinate: 767,567
alignment length: 1,308 bp

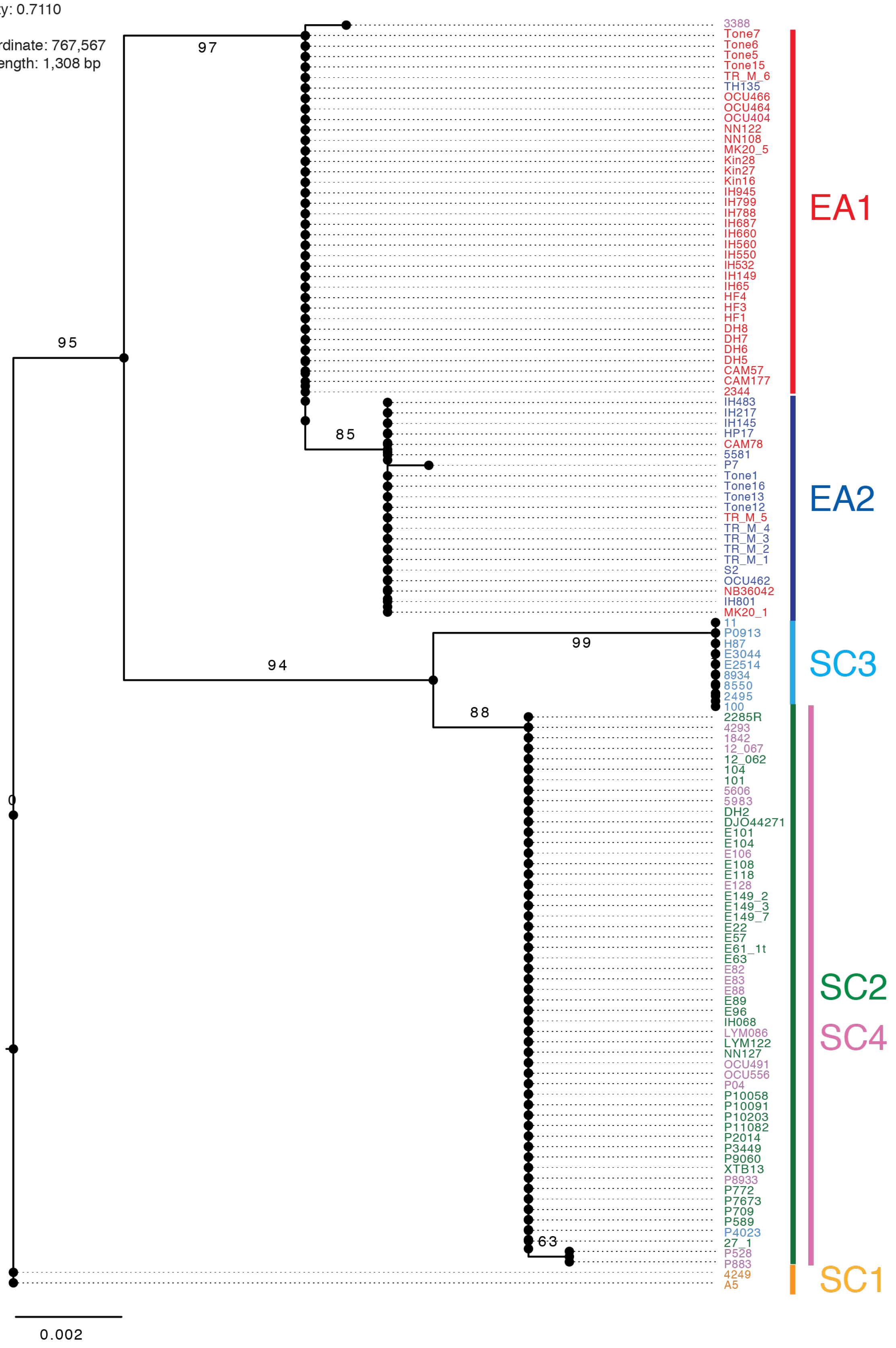

Additional file 3

(iv) MAH_0809

locus_tag: MAH_0809 in strain TH135; MAV_0960 in strain 104
gene name: not assigned
product name: hypothetical protein or membrane protein
nuc. diversity: 9.4242
hap. diversity: 0.6985
n.allele: 10
TH135 coordinate: 813,897
alignment length: 758 bp

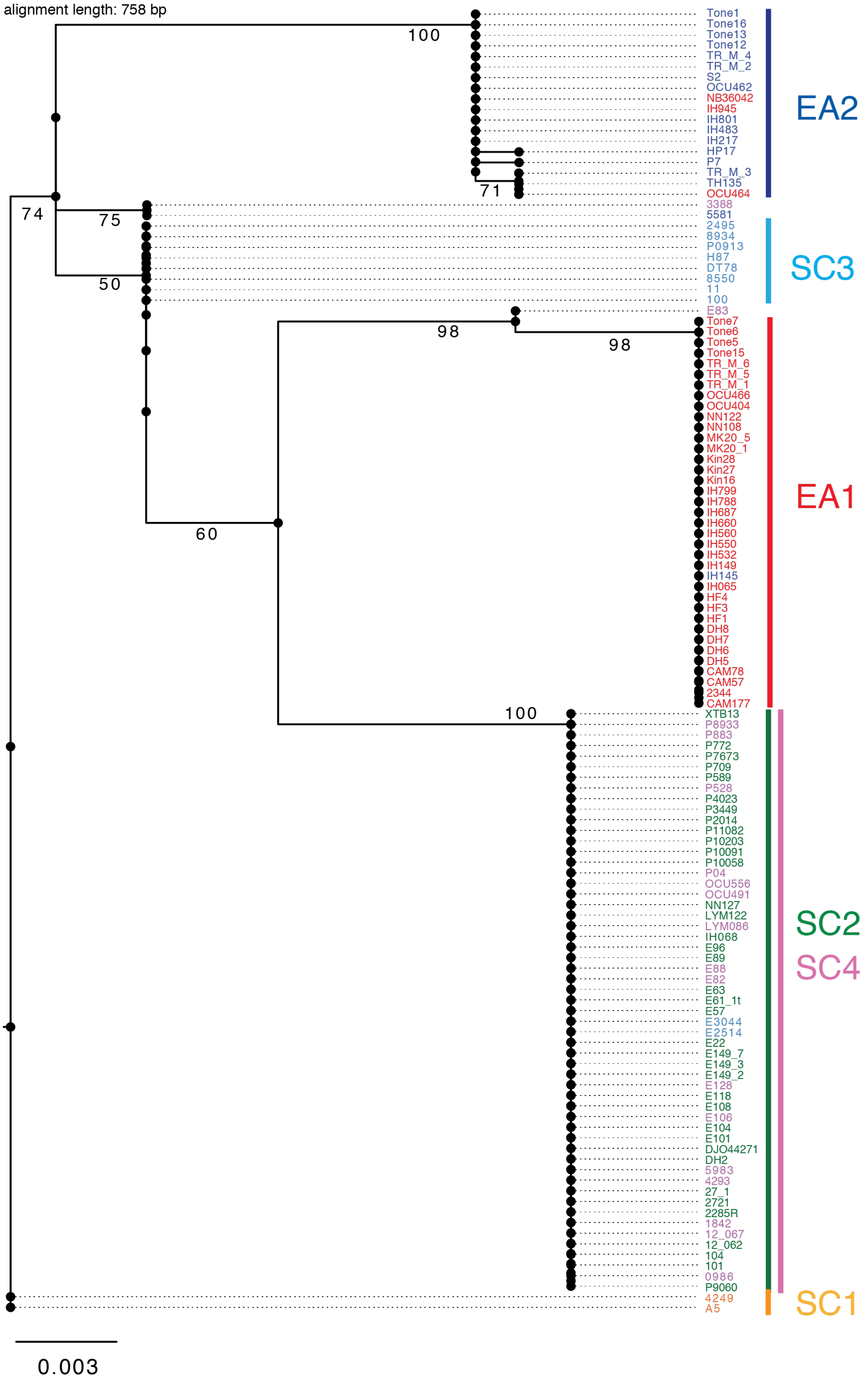

Additional file 3

(v) MAH_2714

locus_tag: MAH_2714 in TH135; MAV_2410 in strain 104
gene name: tatC
product name: Sec-independent protein translocase protein TatC
nuc. diversity: 5.3282
hap. diversity: 0.6716
n.allele: 8
TH135 coordinate: 2,965,182
alignment length: 861 bp

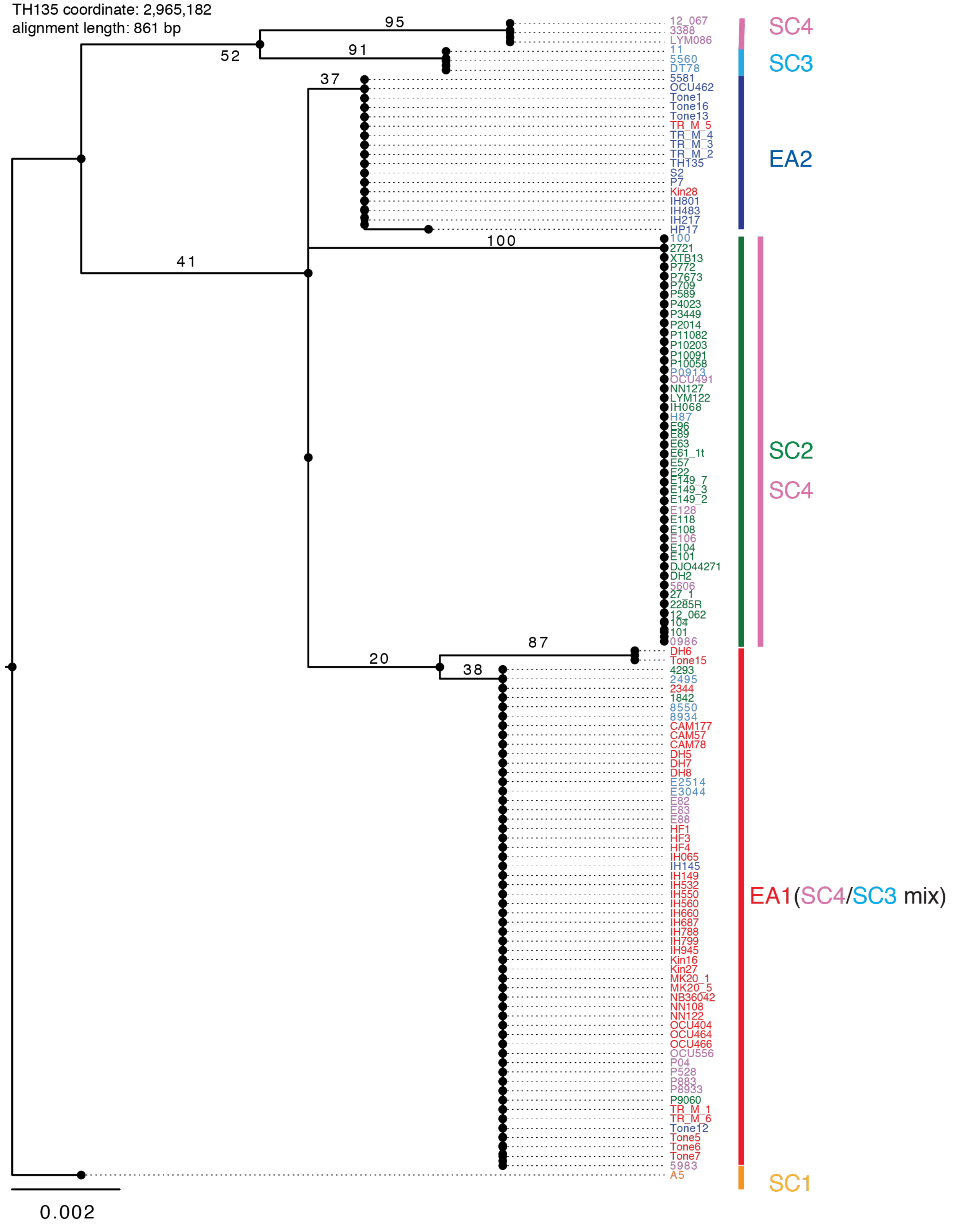

Additional file 3

(vi) *hsp65*

locus tag: MAH_4120 in strain TH135; MAV_4707 in strain 104
gene name: *groEL1*
product name: molecular cheperone GroEL
nuc.diversity: 2.7827
hap.diversity: 0.8262
n.allele: 12
TH135 coordinate: 4,400,278
alignment length: 1,623 bp

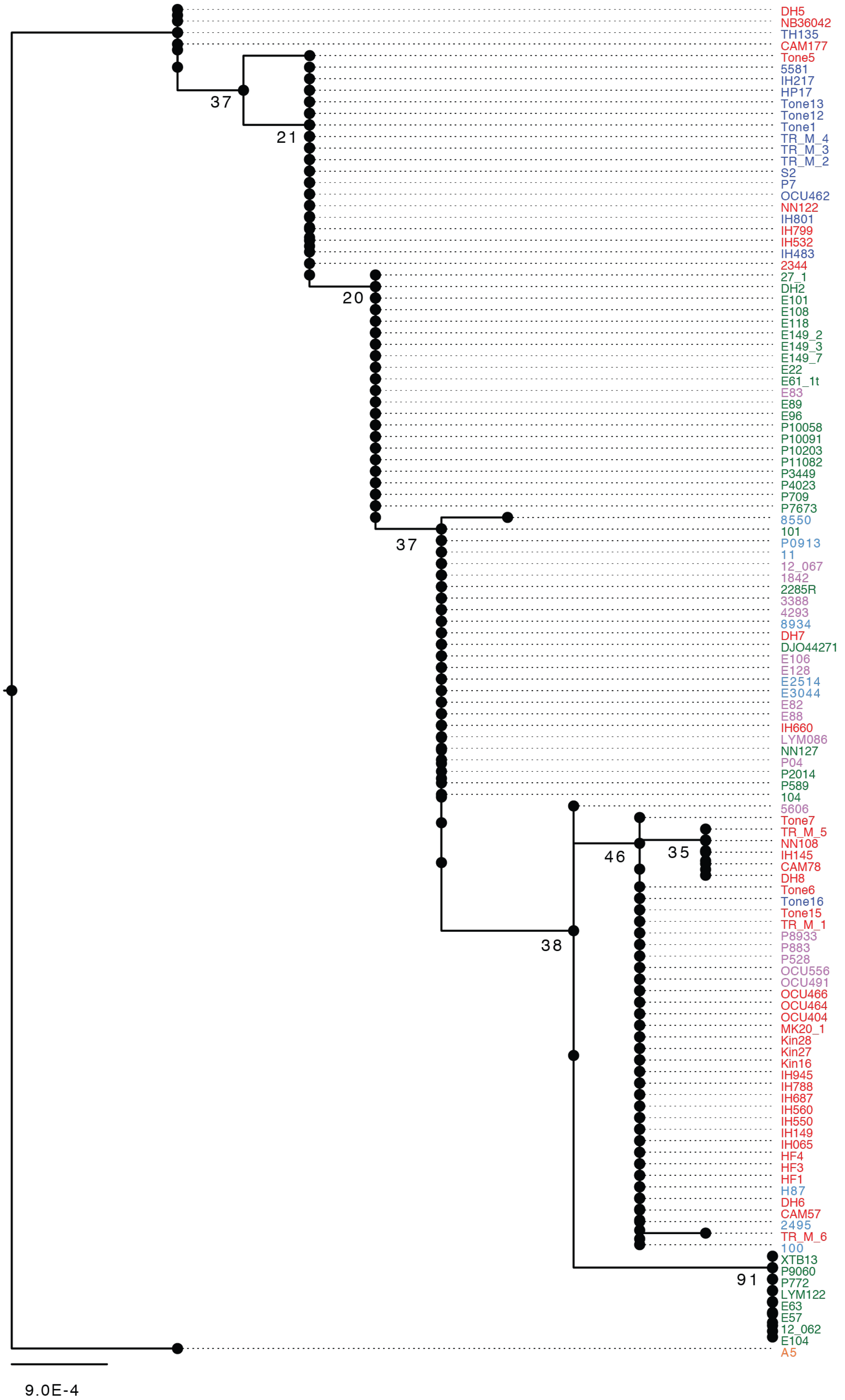

Additional file 3

(vii) MAV_1375

locus tag: MAH_1236 in strain TH135; MAV_1375 in strain 104
gene name: *sugA*
product name: trehalose ABC transporter permease
nuc.diversity: 4.909769
hap.diversity: 0.6967880
n. allele: 9
TH135 coordinate: 1,259,977
alignment length: 876 bp

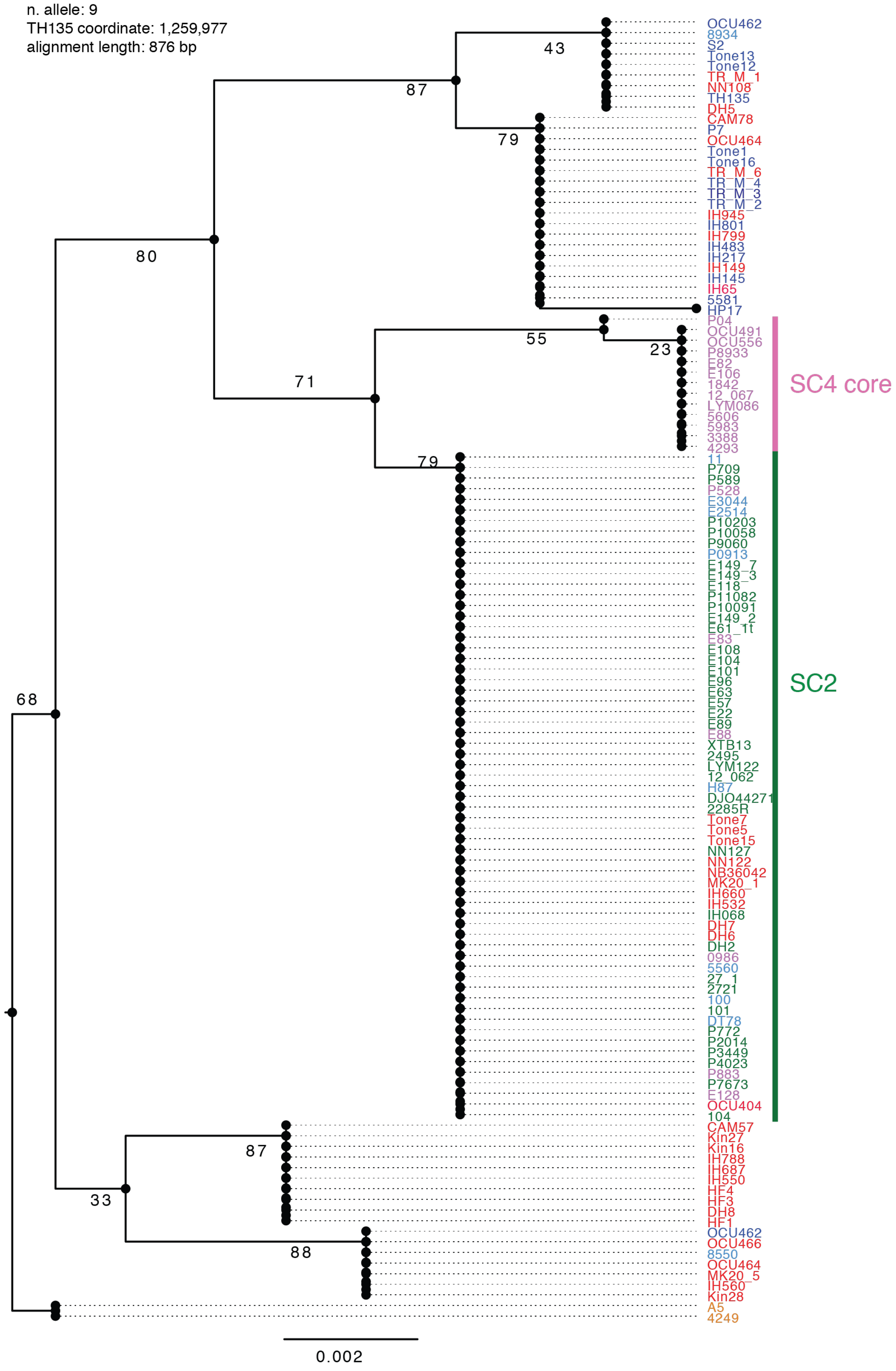

(viii) MAV_2820

locus tag: MAH_2466 in strain TH135; MAV_2820 in strain 104
gene name: not assigned
product name: short-chain dehydrogenase
nuc.diversity: 3.557510
hap.diversity: 0.7974144
n.allele: 12
TH135 coordinate: 2,713,718
alignment length: 612 bp

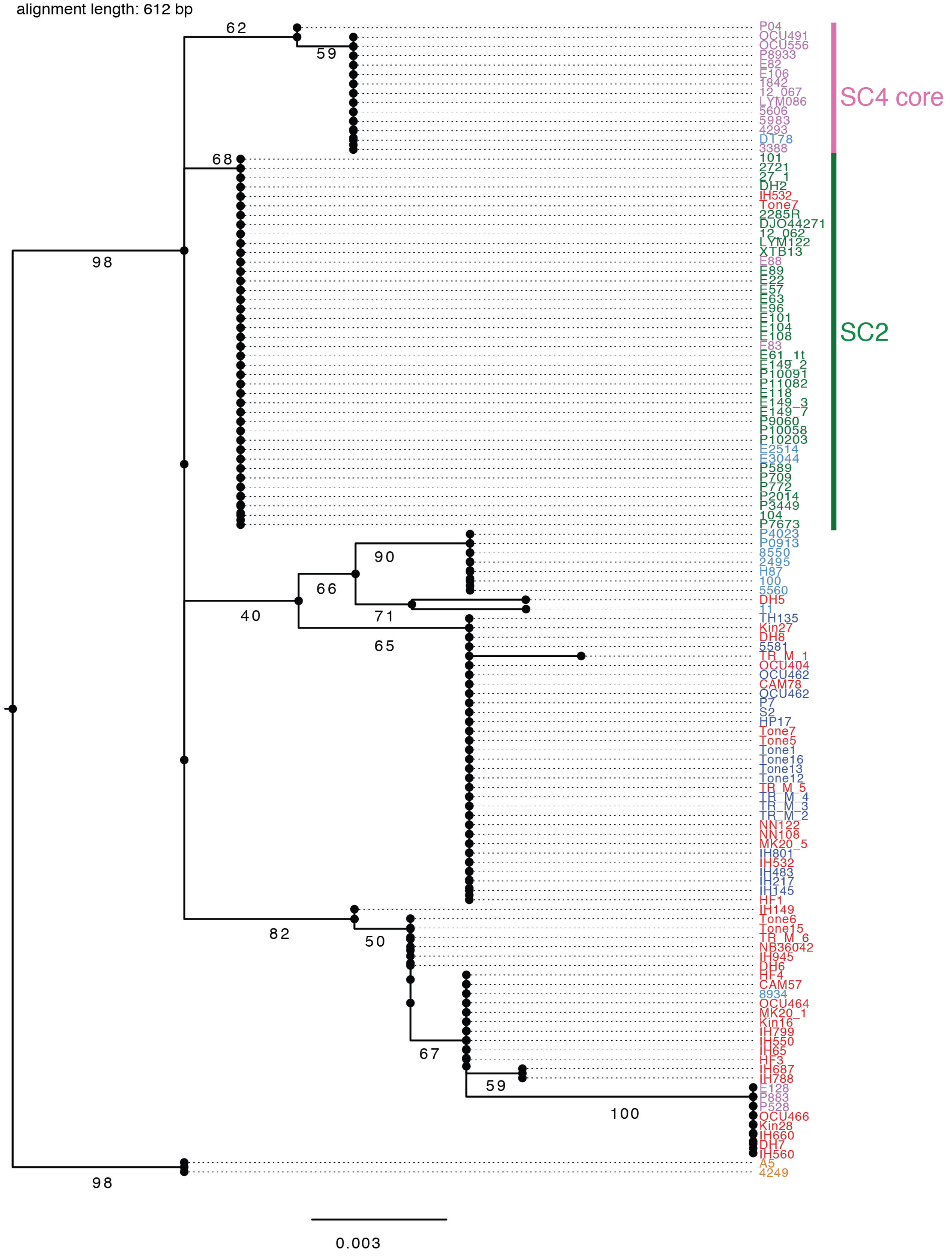

Supplement: Supplementary file 3 — Additional file 3. Phylogenetic trees based on codon alignments of marker gene candidates and hsp65. (i) MAH_0788/MAV_0940 ortholog, (ii) MAH_0771/MAV_0930 ortholog, (iii) MAH_0766/MAV_0925 ortholog, (iv) MAA_0809/MAV_0960 ortholog, (v) MAH_2714/ MAV_2410 ortholog (inadequate marker gene), (vi) hsp65 ortholog, (vii) MAV_1375 ortholog, (viii) MAV_2820 ortholog Trees were constructed using the GTR + G model in PhylML with 100 times bootstrapping run. Values by the branch indicate the number of bootstrap supports. Allele of strain A5 was used as an out group for the analysis in panels i–vi. The out group was not used for tree construction in panels vii and viii. Alignment information is shown by the tree as follows: n.allele, number of alleles in the alignment (population); nuc.diversity, average pairwise nucleotide diversity (number of site differences) in the alignment; hap.diversity, probability of haplotype (allele) differences in the alignment; alignment length, length of alignment without gaps. [file 12864_2019_6078_MOESM3_ESM.pdf]
