# Supplementary material for: The recombination-cold region as an epidemiological marker of recombinogenic opportunistic pathogen Mycobacterium avium
Source: BMC Genomics. 2019 Oct 17;20:752. doi: 10.1186/s12864-019-6078-2 (PMC6798384; doi:10.1186/s12864-019-6078-2)
Supplement: Supplementary file 4 — Additional file 4. Alignment of lineage-specific alleles. (A) MAH_0788/MAV_0940 locus (cinA/P-450 gene). (B) MAH_1236/MAV_1375 locus (sugA gene). Polymorphic sites were indicated by distinct color. Sites used to distinguish among M. avium lineages were indicated by asterisks under the alignment. [file 12864_2019_6078_MOESM4_ESM.pdf]

Additional file 4

A. MAH_0788/MAV_0940

```
Mint(ATCC13950)   1 TTGAGTGTCG ACGACGTCGT CAATGACGGT GACCGGAAGA AGAACCGATA CCACTTCGAT CGGCATTCTT CCGAGTATCG   80
MAS(ATCC49884)    1 TTGAGTGTCG ACGACGGCGT CAATGACAGC GACCGCAAGA AGAACCGGTA TCACTTCGAC CGGCATTCCC CGGACTATCG   80
MAP(K10)          1 ---------- ---------- ---------- ---------- ---------- ---------- ---------- ----------   80
SC2/SC4/MAA       1 TTGAGTGTCG ACGACGGCGT CAATGACAGC GACCGCAAGA AGAACCGGTA TCACTTCGAC CGGCATTCCC CGGACTATCG   80
SC1(A5)           1 TTGAGTGTCG ACGACGGCGT CAATGACAGC GACCGCAAGA AGAACCGGTA TCACTTCGAC CGGCATTCCC CTGACTATCG   80
SC3(H87)          1 TTGAGTGTCG ACGACGGCGT CAATGACAGC GACCGCAAGA AGAACCGGTA TCACTTCGAC CGGCATTCCC CGGACTATCG   80
EA1(Tone5)        1 TTGAGTGTCG ACGACGGCGT CAATGACAGC GACCGCAAGA AGAACCGGTA TCACTTCGAC CGGCATTCCC CGGACTATCG   80
EA2(TH135)        1 TTGAGTGTCG ACGACGGCGT CAATGACAGC GACCGCAAGA AGAACCGGTA TCACTTCGAC CGGCATTCCC CGGACTATCG   80
                                                                                               *
                                                                                               72

Mint(ATCC13950)  81 GTCGCAGTTC AAGGCCATCA CCGAAGAGAT GCACGCCAAG TGCCCCATGG CCTGGACGGA TACCTACGGC GGCCACTGGG  160
MAS(ATCC49884)   81 GTCGCGGTTC AAGGCGATCA CCGAGGAGAT GCACGCGAAG TGCCCGATGG CGTGGACGGA CACCTACGGC GGGCACTGGG  160
MAP(K10)         81 ---------- ---------- ---------- ---------- ---------G CGTGGACGGA CACCTACGGC GGGCACCGGG  160
SC2/SC4/MAA      81 GTCGCGGTTC AAGGCGATCA CCGAGGAGAT GCACGCGAAG TGCCCGATGG CGTGGACGGA CACCTACGGC GGGCACTGGG  160
SC1(A5)          81 GTCGCGGTTC AAGGCGATCA CCGAGGAGAT GCACGCGAAG TGCCCGATGG CGTGGACGGA CACCTACGGC GGGCACTGGG  160
SC3(H87)         81 GTCGCGGTTC AAGGCGATCA CCGAGGAGAT GCACGCGAAG TGCCCGATGG CGTGGACGGA CACCTACGGC GGGCACTGGG  160
EA1(Tone5)       81 GTCGCGGTTC AAGGCGATCA CCGAGGAGAT GCACGCGAAG TGCCCGATGG CGTGGACGGA CACCTACGGC GGGCACTGGG  160
EA2(TH135)       81 GTCGCGGTTC AAGGCGATCA CCGAGGAGAT GCACGCGAAG TGCCCGATGG CGTGGACGGA CACCTACGGC GGGCACTGGG  160
                                                                                                    *
                                                                                                    157

Mint(ATCC13950) 161 TGGCGGCCGG CAGCCACGAG GTCTTCGAGC TGGCGCGCTG CCCGGCGGTC TCCAACGACC ACGACATCCA CGGCGAACGC  240
MAS(ATCC49884)  161 TGGCGGCCGG CAGCCACGAG GTCTTCGAAC TCGCCCGTTG CCCCGCCGTG TCCAACGACC ATGACATCAA CGGCGAACGC  240
MAP(K10)        161 TGGCGGCCGG CAGCCACGAG GTCTTCGAAC TCGCCCGCTG CCCCGCCGTG TCCAACGACC ATGACATCAA CGGCGAACGC  240
SC2/SC4/MAA     161 TGGCGGCCGG CAGCCACGAG GTCTTCGAAC TCGCCCGTTG CCCCGCCGTG TCCAACGACC ATGACATCAA CGGCGAACGC  240
SC1(A5)         161 TGGCGGCCGG CAGCCACGAG GTCTTCGAAC TCGCCCGCTG CCCCGCCGTG TCCAACGACC ATGACATCAA TGGCGAACGC  240
SC3(H87)        161 TGGCGGCCGG CAGCCACGAG GTCTTCGAAC TCGCCCGTTG CCCCGCCGTG TCCAACGACC ATGACATCAA CGGCGAACGT  240
EA1(Tone5)      161 TGGCGGCCGG CAGCCACGAG GTCTTCGAAC TCGCCCGTTG CCCCGCCGTG TCCAACGACC ATGACATCAA CGGCGAACGT  240
EA2(TH135)      161 TGGCGGCCGG CAGCCACGAG GTCTTCGAAC TCGCCCGTTG CCCCGCCGTG TCCAACGACC ATGACATCAA CGGCGAACGC  240
                                                            *                                    *          *
                                                            198                                  231        240

Mint(ATCC13950) 241 CGTGGCTATA AAGGCATTTC GATCCCCACG GCCAGCCGCG TCAGCGCCGT CCGCGGCGGC ATCCTGGAGA TGGACGACCC  320
MAS(ATCC49884)  241 CGTGGCTACA AAGGCATTTC GATCCCCACG GCCAGCCGCG TCAGCGCAGT GCGCGGCGGC ATCCTGGAAA TGGACGACCC  320
MAP(K10)        241 CGTGGCTACA AAGGCATTTC GATCCCGACG GCCAGCCGCG TCAGCGCGGT GCGCGGCGGC ATCCTGGAAA TGGACGACCC  320
SC2/SC4/MAA     241 CGTGGCTACA AAGGCATTTC GATCCCCACG GCCAGCCGCG TCAGCGCAGT GCGCGGCGGC ATCCTGGAAA TGGACGACCC  320
SC1(A5)         241 CGTGGCTACA AAGGCATTTC GATCCCGACG GCCAGCCGCG TCAGCGCGGT GCGCGGCGGC ATCCTGGAAA TGGACGACCC  320
SC3(H87)        241 CGTGGCTACA AAGGCATTTC GATCCCCACG GCCAGCCGCG TCAGCGCGGT GCGCGGCGGC ATCCTGGAAA TGGACGACCC  320
EA1(Tone5)      241 CGTGGCTACA AAGGCATTTC GATCCCCACG GCCAGCCGCG TCAGCGCGGT GCGCGGCGGC ATCCTGGAAA TGGACGACCC  320
EA2(TH135)      241 CGTGGCTACA AAGGCATTTC GATCCCCACG GCCAGCCGCG TCAGCGCAGT GCGCGGCGGC ATCCTGGAAA TGGACGACCC  320
                                                *                     *
                                                267                   288

Mint(ATCC13950) 321 CGAGCACCGC ACCTACCGGA CCGTGCTCAA CCCTTATCTG TCCCCGGCCG CGATCAAGCG CTGGGAGCCG TTCATCGACG  400
MAS(ATCC49884)  321 CGAGCACCGC ATCTATCGCA CGGTGCTCAA CCCGTACCTG TCGCCGGCGG CGGTCAAGCG CTGGGAGCCG TTCATCGACG  400
MAP(K10)        321 CGAGCACCGC ATCTATCGCA CGGTGCTCAA TCCGTACCTG TCGCCGGCGG CGGTCAAGCG CTGGGAGCCG TTCATCGACG  400
SC2/SC4/MAA     321 CGAGCACCGC ATCTATCGCA CGGTGCTCAA CCCGTACCTG TCGCCGGCGG CGGTCAAGCG CTGGGAGCCG TTCATCGACG  400
SC1(A5)         321 CGAGCACCGC ATCTATCGCA CGGTGCTCAA CCCGTACCTG TCGCCGGCGG CGGTCAAGCG CTGGGAGCCG TTCATCGACG  400
SC3(H87)        321 CGAGCACCGC ATCTATCGCA CGGTGCTCAA CCCGTACCTG TCGCCGGCGG CGGTCAAGCG CTGGGAGCCG TTCATCGACG  400
EA1(Tone5)      321 CGAGCACCGC ATCTATCGCA CGGTGCTCAA TCCGTACCTG TCGCCGGCGG CGGTCAAGCG CTGGGAGCCG TTCATCGACG  400
EA2(TH135)      321 CGAGCACCGC ATCTATCGCA CGGTGCTCAA CCCGTACCTG TCGCCGGCGG CGGTCAAGCG CTGGGAGCCG TTCATCGACG  400
                                                 *
                                                 351

Mint(ATCC13950) 401 ACGTGACGCG CGCCTGCCTC GACGAGAAGA TCGAGGGCGG CAGCATCGAC TTCGTCGACG ACCTCGCCAA CATCGTGCCC  480
MAS(ATCC49884)  401 AGGTGACCCG CGCCGCGCTG GACGAGAAGA TCGAAGAGGG CAGCATCGAC TTCGTCGACG ACCTGGCCAA CATCGTGCCG  480
MAP(K10)        401 AGGTGACCCG CGCCGCGCTG GACGAGAAGA TCGAAGAGGG CAGCATCGAC TTCGTCGACG ACCTGGCCAA CATCGTGCCG  480
SC2/SC4/MAA     401 AGGTGACCCG CGCCGCGCTG GACGAGAAGA TCGAAGAGGG CAGCATCGAC TTCGTCGACG ACCTGGCCAA CATCGTGCCG  480
SC1(A5)         401 AGGTGACCCG CGCCGCGCTG GACGAGAAGA TCGAAGAGGG CAGCATCGAC TTCGTCGACG ACCTGGCCAA CATCGTGCCG  480
SC3(H87)        401 AGGTGACCCG CGCCGCGCTG GACGAGAAGA TCGAAGAGGG CAGCATCGAC TTCGTCGACG ACCTGGCCAA CATCGTGCCG  480
EA1(Tone5)      401 AGGTGACCCG CGCCGCGCTG GACGAGAAGA TCGAAGAGGG CAGCATCGAC TTCGTCGACG ACCTGGCCAA CATCGTGCCG  480
EA2(TH135)      401 AGGTGACCCG CGCCGCGCTG GACGAGAAGA TCGAAGAGGG CAGCATCGAC TTCGTCGACG ACCTGGCCAA CATCGTGCCG  480

Mint(ATCC13950) 481 GCCGTCTTCA CCCTGGCCAT GCTCGGCATC CCGCTGAAGA AGTGGAACAT GTACAGCGAG CCGGTGCATG CCGCGGTCTA  560
MAS(ATCC49884)  481 GCCGTCCTGA CGCTGGCGAT GCTGGGCATT CCGCTGAAGA AGTGGAAGAT GTACAGCGAG CCGGTGCACG CCGCGGTCTA  560
MAP(K10)        481 GCCGTCCTGA CGCTGGCGAT GCTGGGCATT CCGCTGAAGA AGTGGAAGAT GTACAGCGAG CCGGTGCACG CCGCGGTCTA  560
SC2/SC4/MAA     481 GCCGTCCTGA CGCTGGCGAT GCTGGGCATT CCGCTGAAGA AGTGGAAGAT GTACAGCGAG CCGGTGCACG CCGCGGTCTA  560
SC1(A5)         481 GCCGTCCTGA CGCTGGCGAT GCTGGGCATT CCGCTGAAGA AGTGGAAGAT GTACAGCGAG CCGGTGCACG CCGCGGTCTA  560
SC3(H87)        481 GCCGTCCTGA CGCTGGCGAT GCTGGGCATT CCGCTGAAGA AGTGGAAGAT GTACAGCGAG CCGGTGCACG CTGCGGTCTA  560
EA1(Tone5)      481 GCCGTCCTGA CGCTGGCGAT GCTGGGCATT CCGCTGAAGA AGTGGAAGAT GTACAGCGAG CCGGTGCACG CCGCGGTCTA  560
EA2(TH135)      481 GCCGTCCTGA CGCTGGCGAT GCTGGGCATT CCGCTGAAGA AGTGGAAGAT GTACAGCGAG CCGGTGCACG CCGCGGTCTA  560
                                                                                                 *
                                                                                                 552
```

Additional file 4

```
Mint(ATCC13950) 561  CACACCCGAG CACTCCCCCG ACATCGAGCG GGTCACCGCG ATGCACCGGG AAATGGGACT CGACATGGTC AACAACATGA  640
MAS(ATCC49884)  561  CACGCCCGAG CACTCTCCCG ACATCGAGCG CGTCACCGCC ATGCACCGCG AGATGGGCCT CGACATGGTC AACAACATGC  640
MAP(K10)        561  CACGCCCGAG CACTCTCCCG ACATCGAGCG CGTCACCGCC ATGCACCGCG AGATGGGCCT CGACATGGTC AACAACATGC  640
SC2/SC4/MAA     561  CACGCCCGAG CACTCTCCCG ACATCGAGCG CGTCACCGCC ATGCACCGCG AGATGGGCCT CGACATGGTC AACAACATGC  640
SC1(A5)         561  CACGCCCGAG CACTCTCCCG ACATCGAGCG CGTCACCGCC ATGCACCGCG AGATGGGCCT CGACATGGTC AACAACATGC  640
SC3(H87)        561  CACGCCCGAG CACTCTCCCG ACATCGAGCG CGTCACCGCC ATGCACCGCG AGATGGGCCT CGACATGGTC AACAACATGC  640
EA1(Tone5)      561  CACCCCCGAA CACTCTCCCG ACATCGAGCG CGTCACCGCC ATGCACCGCG AGATGGGCCT CGACATGGTC AACAACATGC  640
EA2(TH135)      561  CACGCCCGAG CACTCTCCCG ACATCGAGCG CGTCACCGCC ATGCACCGCG AGATGGGCCT CGACATGGTC AACAACATGC  640
                        *     *
                       564   570

Mint(ATCC13950) 641  TCGAGATCCG CGCCAATCCG CGGCCAGGAA TCGTGAACGG TCTGCTCCAG ATGCGCATCG ACGGCGAGCC CGCCCCGGAA  720
MAS(ATCC49884)  641  TCGAGATCCG CGAGAACCCG CGCCCCGGCA TCGTGAATGC GCTGCTGCAG ATGCGCATTG ACGGCGAACC CGCGCCGGAC  720
MAP(K10)        641  TCGAGATCCG CGAGAACCCG CGCCCCGGCA TCGTGAATGC GCTGCTGCAG ATGCGCATTG ACGGCGAACC CGCGCCGGAC  720
SC2/SC4/MAA     641  TCGAGATCCG CGAGAACCCG CGCCCCGGCA TCGTGAATGC GCTGCTGCAG ATGCGCATTG ACGGCGAACC CGCGCCGGAC  720
SC1(A5)         641  TCGAGATCCG CGAGAACCCG CGCCCCGGCA TCGTGAATGC GCTGCTGCAG ATGCGCATTG ACGGCGAACC CGCGCCGGAC  720
SC3(H87)        641  TCGAGATCCG CGAGAACCCG CGCCCCGGCA TCGTGAATGC GCTGCTGCAG ATGCGCATTG ACGGGGAGCC CGCGCCGGAC  720
EA1(Tone5)      641  TCGAGATCCG CGAGAACCCG CGCCCCGGCA TCGTGAATGC GCTGCTGCAG ATGCGCATTG ACGGCGAACC CGCGCCGGAC  720
EA2(TH135)      641  TCGAGATCCG CGAGAACCCG CGCCCCGGCA TCGTGAATGC GCTGCTGCAG ATGCGCATTG ACGGCGAACC CGCGCCGGAC  720
                                                                                  *  *
                                                                                 705 708

Mint(ATCC13950) 721  CTGGAGATCC TCGGCAACCT CGGGTTGGTC ATCGGCGGTG GCTTCGACAC CACGACCGCA CTCACCGCGC ACTCGCTGGA  800
MAS(ATCC49884)  721  CTGGAAATCC TCGGCAACCT GGGGCTGGTC ATCGGCGGCG GGTTCGACAC CACGACCGCC CTGACCGCCC ATTCGCTGGA  800
MAP(K10)        721  CTGGAAATCC TCGGCAACCT GGGGCTGGTC ATCGGCGGCG GGTTCGACAC CACGACCGCC CTGACCGCCC ATTCGCTGGA  800
SC2/SC4/MAA     721  CTGGAAATCC TCGGCAACCT GGGGCTGGTC ATCGGCGGCG GGTTCGACAC CACGACCGCC CTGACCGCCC ATTCGCTGGA  800
SC1(A5)         721  CTGGAAATCC TCGGCAACCT GGGGCTGGTC ATCGGCGGCG GGTTCGACAC CACGACCGCC CTGACCGCCC ATTCGCTGGA  800
SC3(H87)        721  CTGGAAATCC TCGGCAACCT GGGGCTGGTC ATCGGCGGCG GGTTCGACAC CACGACCGCC CTGACCGCCC ATTCGCTGGA  800
EA1(Tone5)      721  CTGGAAATCC TCGGCAACCT GGGGCTGGTC ATCGGCGGCG GGTTCGACAC CACGACCGCC CTGACCGCCC ATTCGCTGGA  800
EA2(TH135)      721  CTGGAAATCC TCGGCAACCT GGGGCTGGTC ATCGGCGGCG GGTTCGACAC CACGACCGCC CTGACCGCCC ATTCGCTGGA  800

Mint(ATCC13950) 801  GTGGCTTTCG GAGCACCCGG AGCAACGGCA GCTGCTCAGC GACGAGCGCA AGACCCTGCT CGACCCGGCG ACCGAAGAGT  880
MAS(ATCC49884)  801  ATGGCTGTCC GAGCACCCCG AACAACGACA GCTGCTCAGC GACGAACGCA AAACGCTGCT CGATCCCGCC ACCGAAGAGT  880
MAP(K10)        801  ATGGCTGTCC GAGCATCCCG AACAACGACA GCTGCTCAGC GACGAACGCA AAACGCTGCT CGATCCCGCC ACCGAAGAGT  880
SC2/SC4/MAA     801  ATGGCTGTCC GAGCATCCCG AACAACGACA GCTGCTCAGC GACGAACGCA AAACGCTGCT CGATCCCGCC ACCGAAGAGT  880
SC1(A5)         801  ATGGCTGTCC GAGCATCCCG AACAACGACA GCTGCTCAGC GACGAACGCA AAACGCTGCT CGATCCCGCC ACCGAAGAGT  880
SC3(H87)        801  ATGGCTGTCC GAGCATCCCG AACAACGACA GCTGCTCAGC GACGAACGCA AAACGCTGCT CGATCCCGCC ACCGAAGAGT  880
EA1(Tone5)      801  ATGGCTGTCC GAGCATCCCG AACAACGACA GCTGCTCAGC GACGAACGCA AAACGCTGCT CGATCCCGCC ACCGAAGAGT  880
EA2(TH135)      801  ATGGCTGTCC GAGCATCCCG AACAACGACA GCTGCTCAGC GACGAACGCA AAACGCTGCT CGATCCCGCC ACCGAAGAGT  880
                                  *
                                 816

Mint(ATCC13950) 881  TCCTGCGCTA CTTCACCCCG GCACCCGGGG ACGGCAGGAC CTTCGCCGAA GACACCGAAC TCGACGGCAC CCGGTTCAAA  960
MAS(ATCC49884)  881  TCCTGCGCTA CTTCACCCCG GCGCCCGGCG ACGGCCGGAC CTTCTCCGAG GACTTCGAAC TCGACGGCAC CGTGTTCAAG  960
MAP(K10)        881  TCCTGCGCTA CTTCACCCCG GCGCCCGGCG ACGGCCGCAC CTTCTCCGAG GACTTCGAAC TCGACGGCAC CGTGTTCAAG  960
SC2/SC4/MAA     881  TCCTGCGCTA CTTCACCCCG GCGCCCGGCG ACGGCCGGAC CTTCTCCGAG GACTTCGAAC TCGACGGCAC CGTGTTCAAG  960
SC1(A5)         881  TCCTGCGCTA CTTCACCCCG GCGCCCGGCG ACGGCCGCAC CTTCTCCGAG GACTTCGAAC TCGACGGCAC CGTGTTCAAG  960
SC3(H87)        881  TCCTGCGTTA CTTCACCCCG GCGCCCGGCG ACGGCCGCAC CTTCTCCGAG GACTTCGAAC TCGACGGCAC CGTGTTCAAG  960
EA1(Tone5)      881  TCCTGCGCTA CTTCACTCCC GCGCCCGGCG ACGGCCGCAC CTTCTCCGAG GACTTCGAAC TCGACGGCAC CGTGTTCAAG  960
EA2(TH135)      881  TCCTGCGCTA CTTCACCCCG GCGCCCGGCG ACGGCCGGAC CTTCTCCGAG GACTTCGAAC TCGACGGCAC CGTGTTCAAG  960
                            *          *                   *
                           888        897                 918

Mint(ATCC13950) 961  GAGGGCGAGC GGCTGTGGAT TTCGTGGGCG ATGGCCAACC GAGACCCCGC GGTGTTCCAC GACCCGGACG AGATCATCCT 1040
MAS(ATCC49884)  961  GAGGGCGAGC GGCTGTGGAT CTCCTGGGCG ATGGCCAACC GCGACCCGGC GGTCTTCCAC GACCCGGACG AGGTGATCCT 1040
MAP(K10)        961  GAGGGCGAGC GGCTGTGGAT CTCCTGGGCG ATGGCCAACC GCGACCCGGC GGTCTTCCAC GACCCGGACG AGGTGATCCT 1040
SC2/SC4/MAA     961  GAGGGCGAGC GGCTGTGGAT CTCCTGGGCG ATGGCCAACC GCGACCCGGC GGTCTTCCAC GACCCGGACG AGGTGATCCT 1040
SC1(A5)         961  GAGGGCGAGC GGCTGTGGAT CTCCTGGGCG ATGGCCAACC GCGACCCGGC GGTCTTCCAC GACCCGGACG AGGTGATCCT 1040
SC3(H87)        961  GAGGGCGAGC GGCTGTGGAT CTCCTGGGCG ATGGCCAACC GCGACCCGGC GGTCTTCCAC GACCCGGACG AGGTGATCCT 1040
EA1(Tone5)      961  GAGGGCGAGC GGCTGTGGAT CTCCTGGGCG ATGGCCAACC GCGACCCGGC GGTCTTCCAC GACCCGGACG AGGTGATCCT 1040
EA2(TH135)      961  GAGGGCGAGC GGCTGTGGAT CTCCTGGGCG ATGGCCAACC GCGACCCGGC GGTCTTCCAC GACCCGGACG AGGTGATCCT 1040

Mint(ATCC13950)1041  CGACCGTAAG GGTAACCGGC ACTTCAGCTT TGGGCTCGGG GTCCACCGAT GCATCGGGTC GAATGTGGCA CGCACGGTGT 1120
MAS(ATCC49884) 1041  CGACCGTAAG GGCAACCGGC ACTTCAGCTT TGGGCTGGGC ATCCACCGCT GCATCGGGTC GAACGTGGCG CGCACCGTGT 1120
MAP(K10)       1041  CGACCGTAAG GGCAACCGGC ACTTCAGCTT TGGGCTCGGC ATCCACCGCT GCATCGGGTC GAACGTGGCG CGCACCGTGT 1120
SC2/SC4/MAA    1041  CGACCGTAAG GGCAACCGGC ACTTCAGCTT TGGGCTGGGC ATCCACCGCT GCATCGGGTC GAACGTGGCG CGCACCGTGT 1120
SC1(A5)        1041  CGACCGTAAG GGCAACCGGC ACTTCAGCTT TGGGCTCGGC ATCCACCGCT GCATCGGGTC GAACGTGGCG CGCACCGTGT 1120
SC3(H87)       1041  CGACCGTAAG GGCAACCGGC ACTTCAGCTT TGGGCTGGGC ATCCACCGCT GCATCGGGTC GAACGTGGCG CGCACCGTGT 1120
EA1(Tone5)     1041  CGACCGTAAG GGCAACCGGC ACTTCAGCTT TGGGCTGGGC ATCCACCGCT GCATCGGGTC GAACGTGGCG CGCACCGTGT 1120
EA2(TH135)     1041  CGACCGTAAG GGCAACCGGC ACTTCAGCTT TGGGCTGGGC ATCCACCGCT GCATCGGGTC GAACGTGGCG CGCACCGTGT 1120
                                                            *
                                                          1077
```

Additional file 4

```
Mint(ATCC13950)1121 TCAAATCCAT GCTGACCGCG GTTCTCGACC GGATGCCCGA CTACAAGTGC GATCCCGAGG GGACCGTCCA TTACGAGACT 1200
MAS(ATCC49884) 1121 TCAAGTCGAT GCTCATCGCG GTGCTCGACC GGATGCCGGA CTACCGCTGC GACCCCGAGG GCACCGTGCA CTACGAGACC 1200
MAP(K10)       1121 TCAAGTCGAT GCTCATCGCC GTGCTCGACC GGATGCCGGA CTACCGCTGC GACCCCGAGG GCACCGTGCA CTACGAGACC 1200
SC2/SC4/MAA    1121 TCAAGTCGAT GCTCATCGCG GTGCTCGACC GGATGCCGGA CTACCGCTGC GACCCCGAGG GCACCGTGCA CTACGAGACC 1200
SC1(A5)        1121 TCAAGTCGAT GCTCATCGCC GTGCTCGACC GGATGCCGGA CTACCGCTGC GACCCCGAGG GCACCGTGCA CTACGAGACC 1200
SC3(H87)       1121 TCAAGTCGAT GCTCATCGCG GTGCTCGACC GGATGCCGGA CTACCGCTGC GACCCCGAGG GCACCGTGCA CTACGAGACC 1200
EA1(Tone5)     1121 TCAAGTCGAT GCTCATCGCG GTGCTCGACC GGATGCCGGA CTACCGCTGC GACCCCGAGG GCACCGTGCA CTACGAGACC 1200
EA2(TH135)     1121 TCAAGTCGAT GCTCATCGCG GTGCTCGACC GGATGCCGGA CTACCGCTGC GACCCCGAGG GCACCGTGCA CTACGAGACC 1200
                                         *
                                       1140

Mint(ATCC13950)1201 ATCGGTGTCA TCCAGGGCAT GCGTAAGCTG CCGGCGACCT TCACGCCCGG CCGCAGGATC GGAGCCGGCC TGGACGAGAC 1280
MAS(ATCC49884) 1201 ATCGGCGTCA TCCAGGGCAT GCGCAAGCTG CCGGCCACAT TCACCCCGGG CCGCCGGATC GGCGCCGGAC TGGACGAGAC 1280
MAP(K10)       1201 ATCGGCGTCA TCCAGGGCAT GCGCAAGCTG CCGGCCACAT TCACCCCGGG CCGCCGGATC GGCGCCGGAC TGGACGAGAC 1280
SC2/SC4/MAA    1201 ATCGGCGTCA TCCAGGGCAT GCGCAAGCTG CCGGCCACAT TCACCCCGGG CCGCCGGATC GGCGCCGGAC TGGACGAGAC 1280
SC1(A5)        1201 ATCGGCGTCA TCCAGGGCAT GCGCAAGCTG CCGGCCACAT TCACCCCGGG CCGCCGGATC GGCGCCGGAC TGGACGAGAC 1280
SC3(H87)       1201 ATCGGCGTCA TCCAGGGCAT GCGCAAGCTG CCGGCCACGT TCACCCCGGG CCGCCGGATC GGCGCCGGGC TGGACGAGAC 1280
EA1(Tone5)     1201 ATCGGCGTCA TCCAGGGCAT GCGCAAGCTG CCGGCCACGT TCACCCCGGG CCGCCGGATC GGCGCCGGAC TGGACGAGAC 1280
EA2(TH135)     1201 ATCGGCGTCA TCCAGGGCAT GCGCAAGCTG CCGGCCACGT TCACCCCGGG CCGCCGGATC GGCGCCGGGC TGGACGAGAC 1280
                                                             *                             *
                                                           1239                          1269

Mint(ATCC13950)1281 GCTCGAAAAG CTCCAACGCA TTTGCGACGA ACAAGAGCTC GCCCGGCCGA TCACCGAACG CAAGGAAGCC GCGGTCATCG 1340
MAS(ATCC49884) 1281 GCTGGAGAAA CTGCAACGCA TCTGCGACGA GCAGGAGCTC GCCCGGCCGA TCACCGAGCG CAAGGAGGCC GCCGTCATCG 1340
MAP(K10)       1281 GCTGGAGAAA CTGCAACGCA TCTGCGACGA GCAGGAGCTC GCCCGGCCGA TCACCGAGCG CAAGGAGGCC GCCGTCATCG 1340
SC2/SC4/MAA    1281 GCTGGAGAAA CTGCAACGCA TCTGCGACGA GCAGGAGCTC GCCCGGCCGA TCACCGAGCG CAAGGAGGCC GCCGTCATCG 1340
SC1(A5)        1281 GCTGGAGAAA CTGCAACGCA TCTGCGACGA GCAGGAGCTC GCCCGGCCGA TCACCGAGCG CAAGGAGGCC GCCGTCATCG 1340
SC3(H87)       1281 GCTGGAGAAA CTGCAACGCA TCTGCGACGA GCAGGAGCTC GCCCGGCCGA TCACCGAGCG CAAGGAGGCC GCCGTCATCG 1340
EA1(Tone5)     1281 GCTGGAGAAA CTGCAACGCA TCTGCGACGA GCAGGAACTC GCCCGGCCGA TCACCGAGCG CAAGGAGGCC GCCGTCATCG 1340
EA2(TH135)     1281 GCTGGAGAAA CTGCAACGCA TCTGCGACGA GCAGGAGCTC GCCCGGCCGA TCACCGAGCG CAAGGAGGCC GCCGTCATCG 1340
                                                       *
                                                     1317

Mint(ATCC13950)1341 AC                                                                                   1342
MAS(ATCC49884) 1341 AC                                                                                   1342
MAP(K10)       1341 AC                                                                                   1342
SC2/SC4/MAA    1341 AC                                                                                   1342
SC1(A5)        1341 AC                                                                                   1342
SC3(H87)       1341 AC                                                                                   1342
EA1(Tone5)     1341 AC                                                                                   1342
EA2(TH135)     1341 AC                                                                                   1342
```

B. MAH_1236/MAV_1375

```
SC2(Mah104)       1 GTGCTGGGCC GGACATCCGA ACAGCGGCTG GCGCTTGTGC TGGTTGCGCC GGCGGCGATT CTGATGCTGG TGGTGACGGC 80
SC4(OCU491)       1 GTGCTGGGCC GGACATCCGA ACAGCGGCTG GCGCTTGTGC TGGTTGCGCC GGCGGCGATT CTGATGCTGG CGGTGACGGC 80
MAA(ATCC25291)    1 GTGCTGGGCC GGACATCCGA ACAGCGGCTG GCGCTTGTGC TGGTTGCGCC GGCGGCGATT CTGATGCTGG CGGTGACGGC 80
                                                                                             *
                                                                                             71

SC2(Mah104)      81 CTACCCGATC GGCTATGCGG TGTGGTTGAG CTTGCAGCGC AACAACCTTG CGGCTCCGCA CGACACCGCG TTCGTCGGCC 160
SC4(OCU491)      81 CTACCCGATC GGCTATGCGG TGTGGTTGAG CTTGCAGCGC AACAACCTTG CGGCTCCGCA CGACACCGCG TTCGTCGGCC 160
MAA(ATCC25291)   81 CTACCCGATC GGCTATGCGG TGTGGTTGAG CTTGCAGCGC AACAACCTTG CGGCTCCGCA CGACACCGCG TTCGTCGGCC 160

SC2(Mah104)     161 TGAGTAACTA CGCGACCATC CTCAGCGACC GGTATTGGTG GACCGCGCTG GCGGTGACGC TGGGCATCAC CGTGGTCTCG 240
SC4(OCU491)     161 TGAGCAATTA CGCGACCATC CTCAGCGACC GGTATTGGTG GACCGCGCTG GCGGTGACGC TGGGCATCAC CGTGGTCTCG 240
MAA(ATCC25291)  161 TGAGTAACTA CGCGACCATC CTCAGCGACC GGTATTGGTG GACCGCGCTG GCGGTGACGC TGGGCATCAC CGTGGTCTCG 240
                        *  *
                      165 168

SC2(Mah104)     241 GTGTCCGCCG AATTCGTGCT GGGCCTGGCG CTGGCGCTGG TGATGCACCG CACCCTGATC GGCAAGGGCC TGGTGCGCAC 320
SC4(OCU491)     241 GTGTCCGCCG AATTCGTGCT GGGCCTGGCG CTGGCGCTGG TGATGCACCG CACCCTGGTC GGCAAGGGCC TGGTGCGCAC 320
MAA(ATCC25291)  241 GTGTCCGCCG AATTCGTGCT GGGCCTGGCG CTGGCGCTGG TGATGCACCG CACCCTGGTC GGCAAGGGCC TGGTGCGCAC 320
                                                                          *
                                                                         298

SC2(Mah104)     321 CGCGGTGCTG ATCCCGTACG GCATCGTCAC CGCGGTCGCG TCCTACAGCT GGTACTACGC CTGGACGCCC GGGACCGGCT 400
SC4(OCU491)     321 CGCGGTGCTG ATCCCGTACG GCATCGTCAC CGCGGTCGCG TCCTACAGCT GGTACTACGC CTGGACGCCC GGAACCGGCT 400
MAA(ATCC25291)  321 CGCGGTGCTG ATCCCGTACG GCATCGTCAC CGCGGTCGCG TCCTACAGCT GGTACTACGC CTGGACGCCC GGGACCGGCT 400
                                                                                              *
                                                                                             393

SC2(Mah104)     401 ATCTGGCCAA CCTGCTGCCG CACGGCAGCG CCCCGCTGAC CGCCCAGATC CCGTCGCTGG CAATCGTCGT GCTCGCCGAG 480
SC4(OCU491)     401 ATCTGGCCAA CCTGCTGCCG CACGGCAGCG CCCCGCTGAC CGCCCAGATC CCGTCGCTGG CAATCGTCGT GCTCGCCGAG 480
MAA(ATCC25291)  401 ATCTGGCCAA CCTGCTGCCG CACGGCAGCG CACCGCTGAC CGCCCAGATC CCGTCGCTGG CAATCGTCGT GCTCGCCGAG 480
                                                  *
                                                 432
```

Additional file 4

```
SC2(Mah104)    481 GTCTGGAAGA CGACGCCGTT CATGTCGCTG CTGCTGCTGG CCGGTCTGGC GCTGGTGCCC GAGGACCTGC TCAAGGCGGC 560
SC4(OCU491)    481 GTCTGGAAGA CGACCCCGTT CATGTCGCTG CTGCTGCTGG CCGGTCTGGC GCTGGTGCCC GAGGACCTGC TCAAGGCGGC 560
MAA(ATCC25291) 481 GTCTGGAAGA CGACCCCGTT CATGTCGCTG CTGCTGCTGG CCGGTCTGGC GCTGGTGCCC GAGGACCTGC TCAAGGCGGC 560
                                  *
                                  495

SC2(Mah104)    561 CCAGGTGGAC GGCGCCGGCG CCTGGCGGCG GCTGACCCGC GTCACCCTGC CGATCATCAA GCCGGCGGTG GTGGTCGCGC 640
SC4(OCU491)    561 CCAGGTGGAC GGCGCCGGCG CCTGGCGGCG GCTGACCCGC GTCACCCTGC CGATCATCAA GCCGGCGGTG GTGGTCGCGC 640
MAA(ATCC25291) 561 CCAGGTGGAC GGCGCCGGCG CCTGGCGGCG GCTGACCCGC GTCACCCTGC CGATCATCAA GCCGGCGGTG GTGGTCGCGC 640


SC2(Mah104)    641 TGTTGTTCCG AACCCTGGAC GCCTTCCGAA TTTTCGACAA CATCTACGTT TTGACCAACG GCGCCAACAA CACCGGTTCG 720
SC4(OCU491)    641 TGTTGTTCCG AACCCTGGAC GCCTTCCGAA TTTTCGACAA CATCTACGTT TTGACCAACG GCGCCAACAA CACCGGTTCG 720
MAA(ATCC25291) 641 TGTTGTTCCG AACACTGGAC GCCTTCCGAA TTTTCGACAA CATCTACGTT TTGACCAACG GCGCCAACAA CACCGGTTCG 720
                                 *
                                 654

SC2(Mah104)    721 GTGTCGATGC TGGGCTACGA CAACCTGTTC AAGGGTTTCA ACGTGGGGCT GGGCTCGGCG ATCAGCGTAC TGATCTTCGG 800
SC4(OCU491)    721 GTGTCGATGC TGGGCTACGA CAACCTGTTC AAGGGTTTCA ACGTGGGGCT GGGCTCGGCG ATCAGCGTGC TGATCTTCGG 800
MAA(ATCC25291) 721 GTGTCGATGC TGGGCTACGA CAACCTGTTC AAGGGTTTCA ACGTGGGGCT GGGCTCGGCG ATCAGCGTGC TGATCTTCGG 800
                                                                                             *
                                                                                             789

SC2(Mah104)    801 ATGCGTGGGC CTGATCGCGC TGGTTTTCGT CAAGGTCTTC GGTGCGGCAG CACCCGGTGG TGACGTCGAT GGCCGT     876
SC4(OCU491)    801 ATGCGTGGGC CTGATCGCGC TGGTTTTCGT CAAGGTCTTC GGTGCGGCAG CCCCCGGTGG TGACGTCGAT GGCCGT     876
MAA(ATCC25291) 801 ATGCGTGGGC CTGATCGCGC TGGTTTTCGT CAAGGTCTTC GGTGCGGCAG CCCCCGGTGG TGACGTCGAT GGCCGT     876
                                                                           *
                                                                           852
```

Alignment of lineage-specific alleles. (A) MAH_0788/MAV_0940 locus (*cinA*/P-450 gene). (B) locus MAH_1236/MAV_1375 (*sugA* gene). Polymorphic sites were indicated by distinct color. Sites used to distinguish among *M. avium* lineages were indicated by asterisks under the alignment.
