# Supplementary material for: The recombination-cold region as an epidemiological marker of recombinogenic opportunistic pathogen Mycobacterium avium
Source: BMC Genomics. 2019 Oct 17;20:752. doi: 10.1186/s12864-019-6078-2 (PMC6798384; doi:10.1186/s12864-019-6078-2)
Supplement: Supplementary file 5 — Additional file 5. Phylogeny inference of SC2 and SC4 members by Gubbins. (Left) Phylogenetic tree based on recombination-tract free alignments. Scale bar indicate the number of SNPs. SC2 members were shown in green, while SC4 members were shown in magenta. (Right) Location of recombination tracts. Recombination tracts introduced in internal braches were shown in red. Recombination tracts unique to terminal branch is shown in blue. (PDF 822 kb) [file 12864_2019_6078_MOESM5_ESM.pdf]

Additional file 5

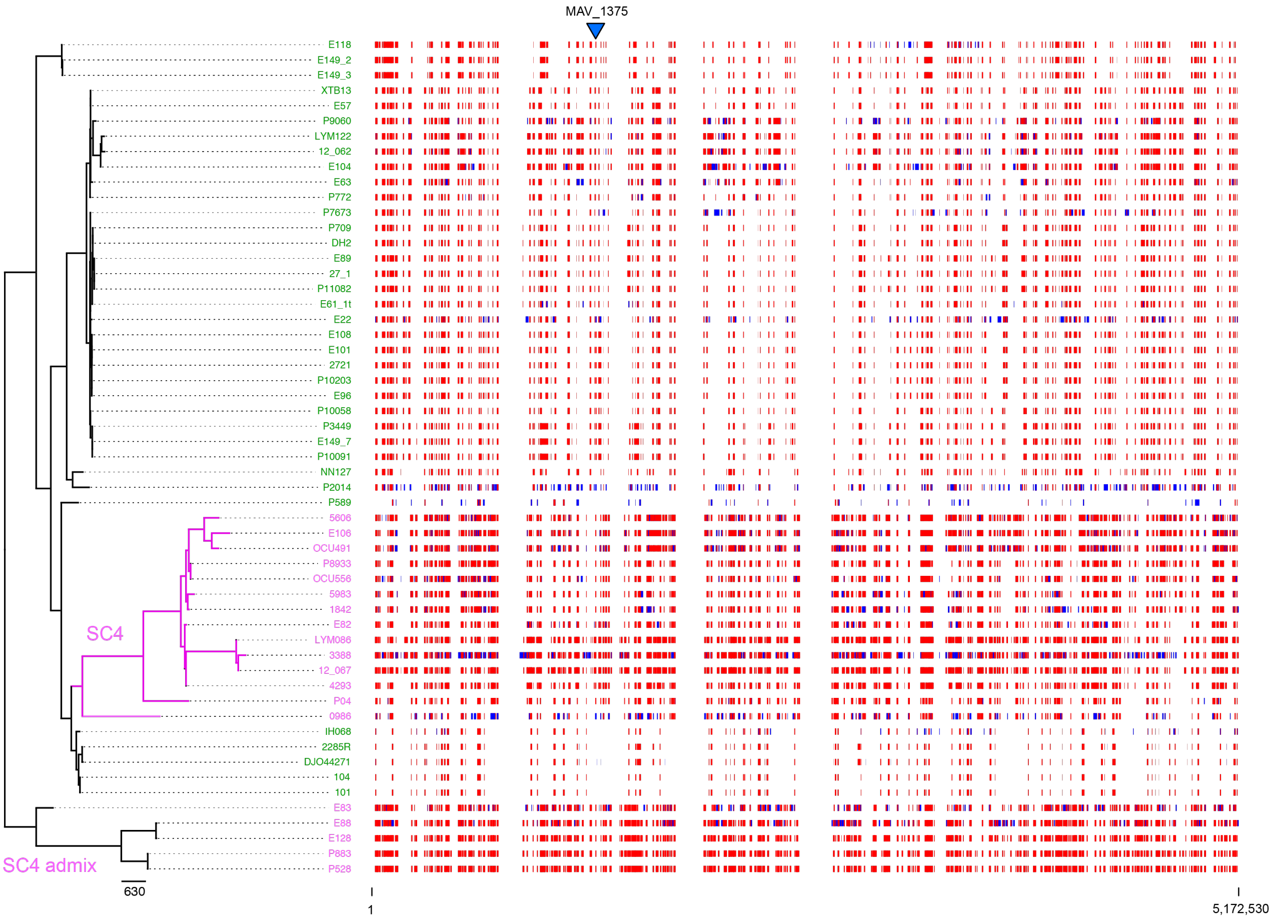

Phylogeny inference of SC2 and SC4 members by Gubbins.
(Left) Phylogenetic tree based on recombination-tract free alignments. Scale bar indicate the number of SNPs. SC2 members were shown in green, while SC4 members were shown in magenta. (Right) Location of recombination tracts. Recombination tracts introduced in the internal branches were shown in red. Recombination tracts unique to terminal branch is shown in blue.
