# Supplementary material for: The recombination-cold region as an epidemiological marker of recombinogenic opportunistic pathogen Mycobacterium avium
Source: BMC Genomics. 2019 Oct 17;20:752. doi: 10.1186/s12864-019-6078-2 (PMC6798384; doi:10.1186/s12864-019-6078-2)
Supplement: Supplementary file 6 — Additional file 6. Insertions near the recombination-cold region. (A) Similarity between two chromosomes. Locations of the recombination-cold region and the MCE operon locus 3 are indicated by white horizontal lines. The similarity between two genomes was determined by the blastn algorithm implemented in GenomeMatcher software [60]. Genomic positions are shown by strain names. (B) Insertions in the tRNA gene cluster. Green pentagons indicate the tRNA gene. Yellow pentagons or circles indicate integrase (a tyrosine recombinase, int) or excisionase (xis). The insert in strain H87 contain 31 copies of recombinase genes. (C) Conservation of marker genes in the MAH recombination-cold region in M. chimaera, M. intracellulare, M. marinum, and M. kansasii. (D) Absence of marker genes in M. abscessus, M. ulcerans, M. tuberculosis, M. canettii, and M. smegmatis. (PDF 7032 kb) [file 12864_2019_6078_MOESM6_ESM.pdf]

The recombination-cold region as an epidemiological marker of recombinogenic opportunistic pathogen *Mycobacterium avium*

Hirokazu Yano[1], Haruo Suzuki[2], Fumito Maruyama[3], Tomotada Iwamoto[4]

1. Graduate School of Life Sciences, Tohoku University. Katahira, Aoba-ku, Sendai, Japan
2. Faculty of environment and information studies, Keio University, Fujisawa, Japan
3. Office of Industry-Academia-Government and Community Collaboration, Hiroshima University, Japan.
4. Kobe Institute of Health, Kobe, Japan

Contents of additional file 6

Insertions near the recombination-cold region.

A. Similarity between two chromosomes. Locations of the recombination-cold region and the MCE operon locus 3 are indicated by white and cyan horizontal lines, respectively. The similarity between two genomes was determined by the blastn algorithm implemented in GenomeMatcher software. Genomic positions are shown by strain names.

B. Insertions in the tRNA gene cluster. Green pentagons indicate the tRNA gene. Yellow pentagons or circles indicate integrase (a tyrosine recombinase, *int*) or excisionase (*xis*). The insert in strain H87 contain 31 copies of recombinase genes.

C. Conservation of marker genes in *M. chimaera*, *M. intracellulare*, *M. marinum*, and *M. kansasii*. 2D comparison plot was made using blastp algorism in GenomeMatcher.

D. Absence of marker genes in *M. abscessus, M. ulcerans, M. tuberculosis*, *M. canettii,* and *M. smegmatis.*

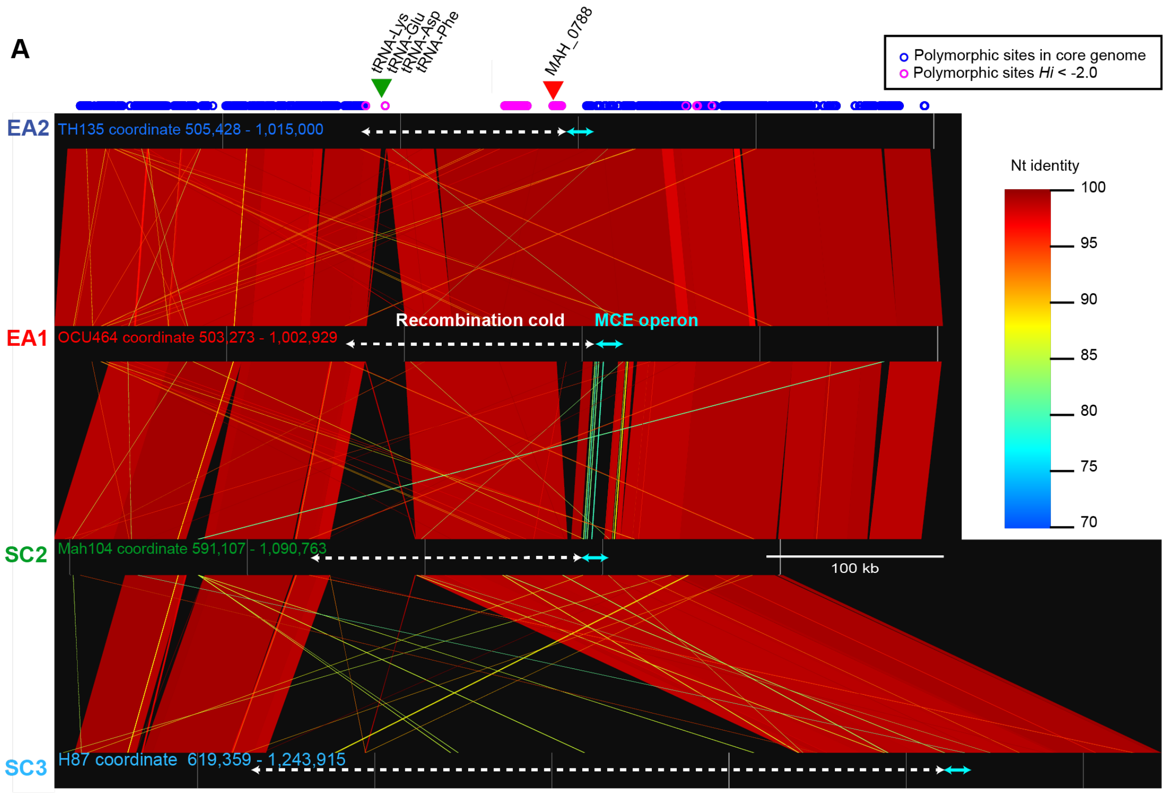

A Similarity between two chromosomes. Locations of the recombination-cold region and the MCE operon locus 3 are indicated by white horizontal lines. The similarity between two genomes was determined by the blastn algorithm implemented in GenomeMatcher software. Genomic positions are shown by strain names.

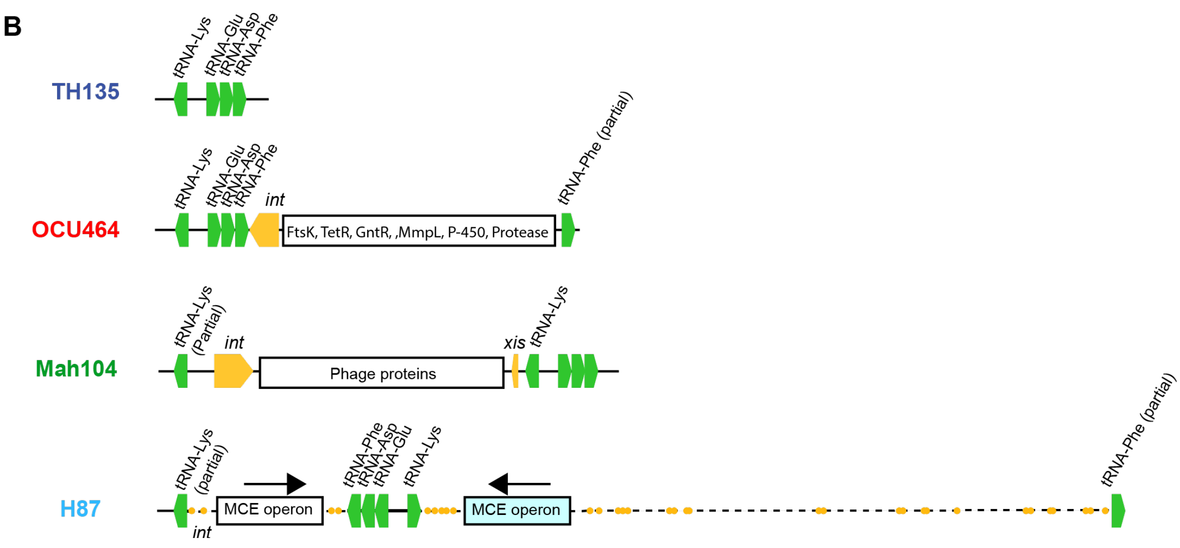

B Insertions in the tRNA gene cluster. Green pentagons indicate the tRNA gene. Yellow pentagons or circles indicate integrase (a tyrosine recombinase, *int*) or excisionase (*xis*). The insert in strain H87 contain 31 copies of recombinase genes.

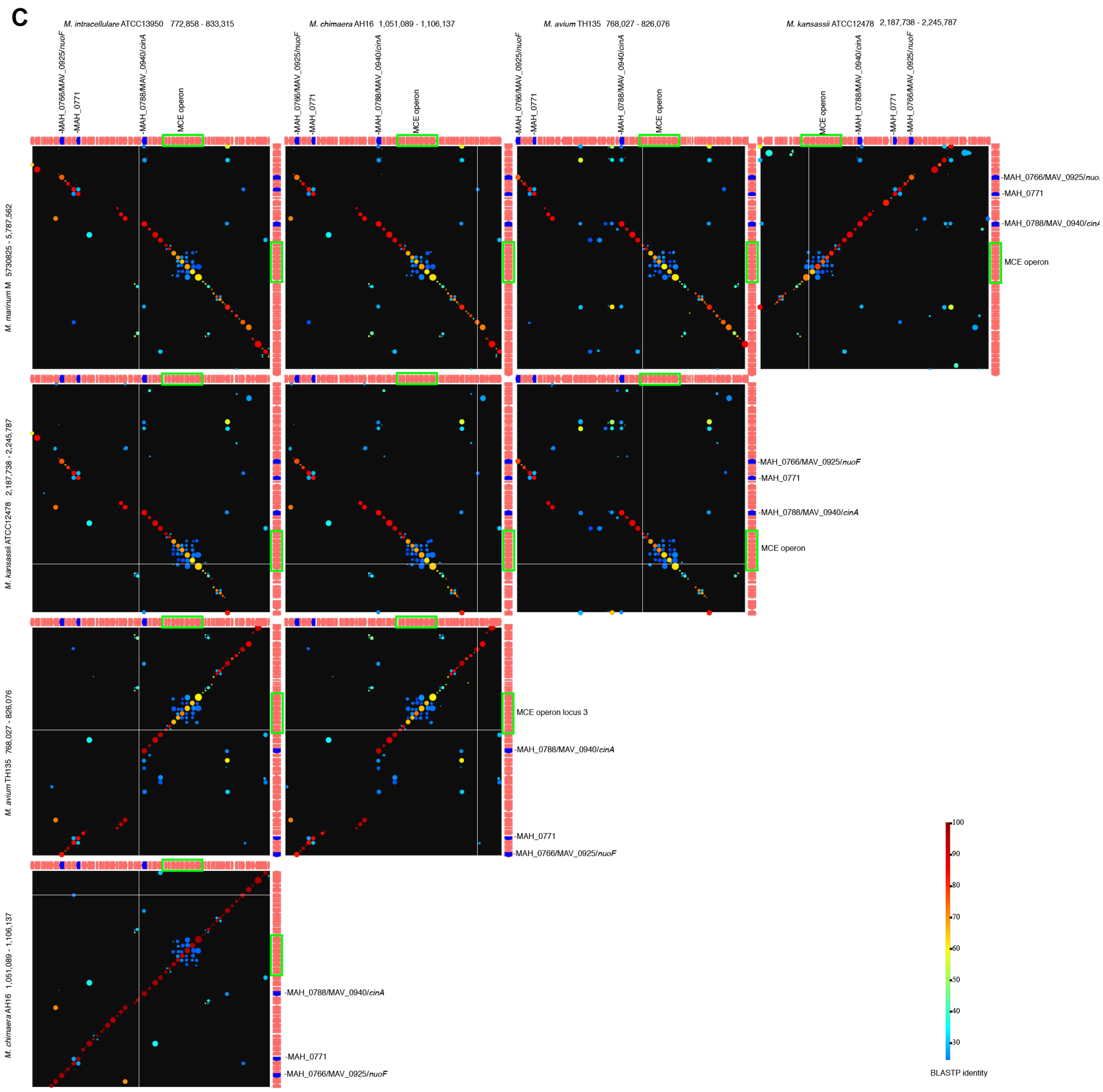

C. Conservation of marker genes in *M. chimaera*, *M. intracellulare*, *M. marinum*, and *M. kansasii*.

Additional file 6

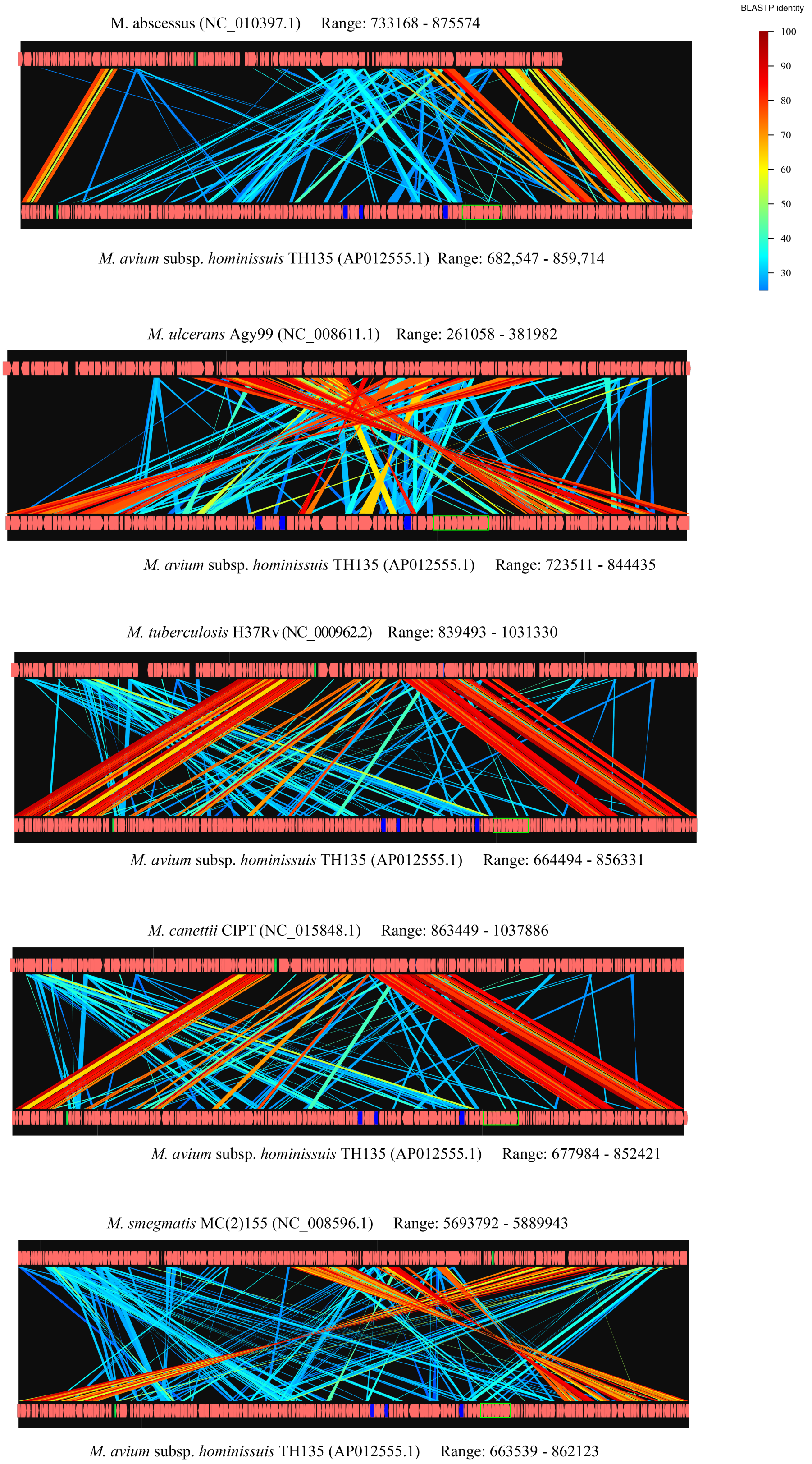

D. Absence of marker genes in *M. abscessus, M. ulcerans, M. tuberculosis*, *M. canettii*, and *M. smegmatis*.
